# A Systematic Review and Meta-Analysis of Mobile Devices and Weight Loss *with an Intervention Content Analysis*

**DOI:** 10.3390/jpm4030311

**Published:** 2014-06-30

**Authors:** Lynnette Nathalie Lyzwinski

**Affiliations:** MPhil Public Health, BA Health Science, Department of Public Health, Cambridge University, Cambridge CB3 OBN, UK; E-Mails: lnl25@cam.ac.uk or Lynnette.lyzwinski@cantab.net

**Keywords:** mobile health, mobile devices, obesity, weight loss, RCTs

## Abstract

Introduction: Overweight and obesity constitute leading global public health challenges. Tackling overweight and obesity by influencing human behaviour is a complex task, requiring novel emerging health psychology interventions. The aims of this review will be to determine whether mobile devices induce weight loss and improvements in diet and physical activity levels when compared with standard controls without a weight loss intervention or controls allocated to non-mobile device weight loss interventions. Methods: A systematic review on mobile devices and weight loss was conducted. The inclusion criteria were all randomized controlled trials with baseline and post-intervention weight measures in adult subjects >18 years of age without pre-specified co-morbidities. Mobile device specifications included modern, portable devices in the form of smartphones, PDAs, iPods, and Mp3 players. Cohen’s d for standardized differences in mean weight loss was calculated. A random effects meta-analysis was generated using Comprehensive meta-analysis software. Theories and intervention content were coded and analysed. Results: A total of 17 studies were identified, of which 12 were primary trials and 5 were secondary analyses. The meta-analysis generated a medium significant effect size of 0.430 (95% CI 0.252–0.609) (*p*-value ≤ 0.01), favouring mobile interventions. Throughout the systematic review, mobile devices were found to induce weight loss relative to baseline weight. When comparing them with standard no intervention controls as well as controls receiving non-mobile weight loss interventions, results favoured mobile devices for weight loss. Reductions in Body mass index, waist circumference, and percentage body fat were also found in the review. Improvements in the determinants of weight loss in the form of improved dietary intake and physical activity levels were also found. Theory appears to largely inform intervention design, with the most common theories being Social Cognitive Theory, Elaboration Likelihood Theory, Control Theory, and Goal Theory. The use of behavioural change techniques was widespread across the studies, with a minimum of five per intervention. Conclusion: Mobile devices appear to induce positive changes in the behavioural determinants of weight and subsequently are associated with weight loss. Mobile device interventions are heavily informed by theory and behaviour change techniques. The use of theory appears to effectively enhance levels of constructs targeted by interventions.

**Table of Content**
Table of Content3121. Introduction3151.1. Background and Epidemiology3151.2. Morbidity and Mortality3161.3. Economic Costs3161.4. Global Strategies3161.5. Diet and Physical Activity Targets3161.6. Health Psychology317Health Psychology Theories3171.7. Behaviour Change Techniques3171.8. Mobile Technology Definition3181.9. Mobile Technology Usage and Public Health Applications3181.10. Research on Mobile Devices and Weight Loss3181.11. Aims and Research Questions3192. Methods3202.1. Overview3202.2. Databases Searched3202.3. Search Limiters3202.4. Search Terminology3202.5. Inclusion and Exclusion Criteria3212.6. Data Extraction3222.7. Study Quality Assessment3222.8. Data Coding3222.9. Data Synthesis3222.10. Data Analysis3233. Results Part A: Systematic Review with Meta-Analysis3233.1. General Search Results3233.2. General Descriptive3233.3. Mobile Device Intervention Media3243.4. Target Behaviour and Weight Loss3243.5. Dietary Measures3433.6. Dietary Changes Overview3433.6.1. Dietary Changes in Fruit and Vegetable Intake3433.6.2. Dietary Changes in Sugar and Fat Intake3443.6.3. Dietary Changes in Daily Caloric Intake3443.6.4. Changes in EBI and ED Scores3443.7. Physical Activity Measures3453.8. Physical Activity Overview3453.8.1. Perceived Physical Activity Goal Adherence3453.8.2. Changes in Physical Activity Levels3453.9. Weight Measures3463.10. Weight Loss Overview3463.10.1. Changes in Weight Mobile Phones3463.10.2. Changes in Weight Other Mobile Devices3473.10.3. Weight Loss and Adherence3473.11. Changes in BMI3473.12. Changes in Waist Circumference3473.13. Changes in Body Fat Percentage3483.14. Study Quality3483.15. Risk of Bias Grading3493.16. Meta-Analysis Weight Loss (kg)3533.16.1. Overview3533.16.2. Results3533.16.3. Heterogeneity3533.16.4. Publication Bias3543.16.5. Sensitivity Analysis3544. Results Part B: Intervention Content Analysis; Use of Theory and Behavior Change Techniques3554.1. Theoretical Base3554.2. Predictors/Constructs3564.2.1. Intentions and Sense of Control3564.2.2. Positive Affect3564.2.3. Self-Efficacy3564.2.4. Elaboration and Reduced Cognitive Load3564.3. Intervention Components3574.3.1. Text Message and App Component3574.3.2. Health Education Component3574.3.3. Professional Support Component3574.3.4. Web Component3624.3.5. Technological Components3624.3.6. Comparator3624.4. Behaviour Change Techniques3624.4.1. Goal Setting, Self-Monitoring and Feedback3624.4.2. Social Support3634.4.3. Prompt Practice3634.4.4. Stress Management and Relapse Prevention3634.4.5. Graded Tasks3634.4.6. Modelling/Demonstrating behaviour3634.4.7. Social Comparison3634.4.8. Barrier Identification3644.4.9. Provision of Encouragement3644.4.10. Contingent Awards3644.4.11. Prompt Intention Formation3644.4.12. Follow-Up Prompts3644.4.13. Provide Instructions3644.4.14. Prompt Practice3645. Discussion Part A: Implications of Mobile Device Interventions for Weight Loss3655.1. Changes in Weight3655.2. Changes in BMI, Body Fat Percentage, and Waist Circumference3655.3. Changes in Diet and Physical Activity Levels3665.4. Intervention Feature Complexity3665.5. Clinical Significance3665.6. Implications of Negative Findings3675.7. Importance of Comparator3676. Discussion Part B: The Implications of Theory and Behaviour Change Techniques3676.1. Theory3676.2. Predictors3706.3. Interaction with Predictors3706.4. Research on Physiological Pathways3706.5. Applied Theories Informing Intervention Design3706.5.1. Common Theories3706.5.2. Less Frequent Theories3716.5.3. Implicit Theory3716.6. Behaviour Change Techniques3726.6.1. Key Adopted Behaviour Change Techniques3726.6.2. Diverse Media of BCT Delivery3756.7. Connection of Behavioural Change Techniques with Theory3757. Summary of Discussions Part A and B3767.1. Synopsis3767.2. Strengths and Limitations3777.2.1. Strengths3777.2.2. Limitations3777.3. Future Directions3788. Conclusions3788.1. Primary Central Research Objective3788.2. Secondary Research Objective379Acknowledgements380Conflicts of Interest380References381Appendix384


**Tables and Figures**
Figure 1. Flow Chart of Search325Table 1. Study Characteristics of Mobile Phone Interventions326Table 2. Critical Appraisal Trial Quality Rating350Table 3. Risk of Bias Grading , Adapted from the Cochrane Hanbook Higgins *et al*352 Figure 2. Mobile Devices and Weight Loss Meta-analysis 353 Figure 3. Funnel Plot for Publication Bias354 Figure 4. Sensitivity Analysis355 Table 4. Intervention Components358Table 5. Theory adapted from Michie and Prestwich Theory Coding and Michie and Abraham Illustrative Theory Techniques368Table 6. Application of Abraham and Michie et al. (2007) 26 Item Coding Manual for Behaviour Change Techniques373Figure 5. BCT and Theory Connection in Reviewed Trials376 Table A1. Summary of CINAHL Search via EbscoHost384


## 1. Introduction

### 1.1. Background and Epidemiology

Obesity and overweight constitute leading global public health challenges of the 21st Century. They have transcended national boundaries to a scale requiring cross-national collaboration for the promulgation of effective global public health policy and population wide interventions. Obesity refers to a Body Mass Index over 30 kg/m^2^ and overweight a BMI over 25 kg/m^2^, measured as a ratio of weight in kg over height in meters squared [1]. According to the WHO [1], obesity has nearly doubled over the past three decades, with over 11% of the world’s population being obese in 2008 [1]. A total of 200 million men and 300 million women age 20 and over were obese by the year 2008, with global estimates by the WHO of the overweight pandemic reaching 1.4 billion [1]. Mathematical modelling projections estimate that under current trends, there will be a total of 2.16 billion overweight and 1.12 billion obese individuals across the globe by the year 2030 [2].

### 1.2. Morbidity and Mortality

Overweight and obesity increase the risk of premature morbidity and mortality. The WHO estimates that approximately 3 million annual deaths are attributed to overweight and obesity [1]. Additionally, they increase the risk of leading chronic diseases including cancer, diabetes, and cardiovascular disease according to the WHO [1]. The attributable risk due to overweight and obesity is 7%–10% for cancer, 44% for diabetes, and 7% for CVD according to WHO estimates [1]. Findings in the Global Burden of Disease Report indicate that a high BMI has increased as a leading risk factor between 1990 and 2010 from the 11th position to being the 6th global risk factor for men and women [3]. The leading causes of obesity and overweight, an unhealthy diet and physical inactivity [1], have also increased as leading global risk factors between 1990 to 2010 [3]. Approximately 2.8% of all deaths worldwide are attributed to low fruit and vegetable intake [4]. A total of 6% of global deaths are attributed to physical inactivity [5].

### 1.3. Economic Costs

In addition to the significant impact on morbidity and mortality, obesity and overweight pose significant economic burdens on nations. Global estimates of the costs of obesity in proportion to total healthcare expenditures are 0.7%–2.8%, with medical costs among obese being 30% higher than in the non-obese population [6]. Under present trends, obesity is estimated to cost the National Health Service in England 6.7 billion by the year 2050 [7].

### 1.4. Global Strategies

Given the significant public health and economic burdens associated with obesity and overweight, it has been placed on the forefront of the health policy agenda. The Political Declaration of the High Level Meeting of the United Nations General Assembly on the Prevention and Control of Non-Communicable Diseases on September 2011 established a precedent for a global political commitment to enforce the determinants of health and to capitalize upon the 2004 WHO Global Strategy on Diet, Physical Activity, and Health [1].

### 1.5. Diet and Physical Activity Targets

Present global obesity strategies aim to target lifestyle choices in the form of healthy eating and physical activity at the population level [8]. The WHO global physical activity guidelines recommend that adults engage in at least 150 min of moderate to vigorous intensity activity per week [9]. There should be bouts of aerobic activity, which increases the heart and breathing rate for a minimum of 10 min, which may be replaced by 75 min of vigorous intensity aerobic activity per week [9]. The WHO Global Obesity Strategy for Diet aims to encourage populations to reduce their intake of saturated fats and trans-fatty acids, sugar, sodium, and increase the consumption of fruit and vegetables [4]. The target intake levels are 400 grams of fruit and vegetables a day [4]. According to the UK Food Standard Agency, trans fatty acids intake should not exceed 2% of total daily food energy, mono saturated fat intake should not exceed 13% of total daily food energy, total fat intake should not exceed 35% of daily food energy, and sugar intake should not exceed 11% of total daily food energy [10]. Adult daily sodium intake should not exceed 6 grams/day [10].

### 1.6. Health Psychology

The fields of behavioural science and health psychology have been actively researching ways to tackle the behavioural determinants of obesity and overweight. Tackling obesity and overweight by changing population health behaviour towards increasing physical activity levels and improving dietary habits is a difficult task requiring novel interventions that target underlying psychological beliefs and processes. According to Webb, behaviours are classified as addictive if they contain a reward-seeking element to them which prevents a given subject’s self-regulatory inhibitory mechanisms from refraining from the behaviour and if the behaviour leads to negative repercussions for the individual [11]. Research suggests that that compulsive unhealthy eating is addictive and is associated with dopamine release and that obese individuals may benefit from similar psychological behavioural treatments as individuals suffering from substance addiction [12]. Given that an unhealthy diet may be classified as addictive makes changing population health behaviour challenging. Research by Tones and Green (1994) suggests that while communication of simple health information to the public is a relatively easy task, changing human behaviour by seeking to alter deeply ingrained attitudes leading to health behaviour change is increasingly difficult [13].

#### Health Psychology Theories

Given the inherent complexity of health behaviours, numerous health psychology theories have been developed to understand ways to change human health behaviour. Well known theories include the Theory of Planned Behaviour, which aims to identify proximal determinants of behaviour change such as intentions towards behavioural change, which may be targeted by interventions [11]. The Transtheoretical Model of Behaviour Change is founded on the premise that individuals undergo 5 stages of change through pre-contemplation, contemplation, preparation, action, and maintenance and medical practitioners may tailor interventions in accordance with patient progress through these stages [14]. Zimmerman *et al.* argue that changing health behaviour including behaviours related to obesity is not a result of a singular decision leading to change in a linear pathway, rather patients often cycle through phases of relapse [14]. Webb argues that health psychology theories geared to change behaviour are complex and variable as they seek to target diverse dimensions of behaviour change including intentions, actions, and relapse prevention [11].

### 1.7. Behaviour Change Techniques

In recognition of the need to develop psychological techniques that target theoretical constructs for behaviour change, Michie and Abraham developed a behaviour change theory coding scheme with a total of 26 possible behaviour change techniques [15,16]. The most actively researched behaviour change technique targeting the behavioural determinants of obesity has been self-monitoring [17]. Furthermore, research by Michie *et al.* [18] has demonstrated that the behaviour change technique of self-monitoring, when combined with at least another behaviour change technique such as goal setting or feedback for instance, increased the effectiveness of interventions aiming to improve physical activity levels and healthy eating. Although self-monitoring with feedback appears to be feasible for weight loss, research suggests that the media through which these techniques for weight loss are delivered may influence the success of the weight loss interventions [19]. That is, traditional interventions have focused on paper media for self-monitoring and Coons *et al.* [19] postulate that mobile methods of self-monitoring may be more effective due to their portability, reach, accessibility, and convenience.

### 1.8. Mobile Technology Definition

Mobile technology refers to portable electronic technology which serves as a medium for communication through transmission and reception of information. It includes different versions of mobile phones and handheld tablets such as personal digital assistants and the new generation tablets such as the iPad [20].

### 1.9. Mobile Technology Usage and Public Health Applications

Mobile technology has increasingly been recognized as a platform for behaviour change interventions. An inherent benefit of mobile devices for health behaviour change interventions is that mobile devices are widely used across the globe, enabling accessibility and scalability of behaviour change interventions at the population level [20]. Global statistics on ownership and usage of mobile devices indicate that there were 5.3 billion cellular phone owners in the year 2010 and that between the years 2007–2010 approximately 200,000 text messages were sent every second [21]. Lefebre [20] ascertains that mobile devices are the future of public health promotion interventions by alluding to the social marketing mix of the price, product, and place convenience of these technologies which enable professional medical support at any time and place, health education, and behavioural self-monitoring [20].

### 1.10. Research on Mobile Devices and Weight Loss

Research on mobile devices has largely focused on their application for smoking cessation. A recent Cochrane meta-analysis found that mobile phone interventions improve smoking quit rates [22]. To date, there has not been any updated systematic review on the latest mobile devices for weight loss with a meta-analysis. There has only been one early systematic review on mobile devices and weight loss and it had positive findings [23]. Since this review, several research papers on emerging modern mobile technology and applications have been published over the three year period. In addition to this, there has been one systematic review on text messaging for weight loss [24]. The only updated systematic review examining all modern mobile devices with a meta-analysis focused on physical activity [25]. The researchers found that mobile device interventions are associated with improvements in physical activity levels [25]. There has not been an in depth review of the most modern mobile devices for weight loss. This review will add to the growing literature on mobile devices and weight loss by reviewing in detail the latest mobile technology for weight loss including mobile apps, text messages, newer PDAs, tablet devices, and MP3 devices.

Second, there has not been any systematic review on the key behavioural change techniques and health psychology theories associated with weight loss by mobile devices. Recently, there has been a new publication on behavioural change techniques for physical activity, but not weight loss [26].

Gaining an updated understanding of the potential of new portable devices to induce weight loss by conducting an updated systematic review and meta-analysis is informative to public health promotion research.

Third, understanding not only whether mobile devices work to reduce weight but also what aspects of these interventions and behaviour change techniques inform successful interventions is important and directly informative to health promotion and health psychology research. According to Michie *et al.* recent CONSORT guidelines for trials require clear descriptions of intervention content in behavioural change research [15]. The Researchers Michie *et al.* ascertain that is crucial to be cognizant of intervention components in order to understand which constituents influence efficacy and to maximize reproducibility in trials [15]. Thus, gaining a greater understanding of the behaviour change techniques as well as components utilized in mobile device weight loss interventions is directly informative to health promotion intervention research in this field.

Furthermore, in recognition of the need to understand the extent to which theory informs health promotion intervention design, Michie and Prestwich (2010) have developed a health psychology theory coding scheme [27]. The researchers argue that often systematic reviews conclude that interventions are theory based, without examining the extent to which health psychology theory truly informs interventions. They also argue that often theory is mentioned in a study, but there is insufficient information to understand how theory informed the intervention and whether the mediators along the causal pathway targeted by the intervention were improved post-intervention. Improvements in cognitive mediators along the causal pathway indicate that the theory was successful in informing the intervention design and targets [27].

Thus, gaining a greater understanding of theory in the field of obesity is needed in order to understand the extent to which theory informs mobile device weight loss intervention design, to understand the predictors the intervention targets and whether they improve post-intervention, and to examine whether certain techniques associated with a given theory are more informative for behaviour change leading to weight loss by mobile device.

### 1.11. Aims and Research Questions

The primary aim of this research will be to provide an updated systematic review and meta-analysis of randomized controlled trials and emerging mobile devices for weight loss. This research will seek to determine whether interventions by mobile devices are effective remedies for obesity and overweight by examining whether they induce weight loss and reductions in body mass index, waist circumference, and body fat percentage. Unlike the early review [23], this research will focus exclusively on modern relatively lightweight portable devices in the form of smartphones such as Blackberries, iPhones, mobile weight loss apps, iPods, MP3 players, and new handheld tablets such as iPads. This research will also examine changes in dietary and physical activity behavioural determinants of weight loss associated with mobile device interventions.

The secondary aim will be to gain a greater understanding of the key underlying health psychology intervention techniques and health psychology theories which target cognitive mediators along the causal pathway to weight loss. This work will involve an adoption of a strong health psychology perspective and theoretical analysis of techniques used, their relationships, and constructs targeted.

*The Central Primary Research Question*: Do mobile devices induce weight loss and favourable changes in diet and physical activity when compared to baseline weight and scores? Do they induce weight loss when compared with standard controls receiving no intervention and or when compared with controls receiving non-mobile weight loss interventions? 

*The Secondary Research Question*: What health psychology theories and psychological behaviour change techniques inform mobile device weight loss intervention design and are theoretical predictors along the causal pathway leading to weight loss improved post-intervention? 

## 2. Methods

### 2.1. Overview

A systematic review and meta-analysis of randomized controlled trials for weight loss and mobile devices was conducted.

### 2.2. Databases Searched

Databases were searched for all RCT’s on weight loss and mobile devices published until May 2013. Databases searched included PubMed (Medline), Google Scholar, CINAHAL, and the Cochrane library. The databases specified on CINAHL Plus included Psych Info, Psyc Articles, and Information Library Science and Technology.

### 2.3. Search Limiters

Limiters were set on CINAHAL for language, peer reviewed journal type, >18 years of age, full references, and text availability in order to specify the search. Limits on the Cochrane database were placed on trials to specify the search.

### 2.4. Search Terminology

Search strings were categorized according to mobile device type and outcome of weight loss. The following search terms were entered into the search engines for mobile devices: (1) ((Text message) or (short message service) or (Multi Media Message Service) or SMS and (Smartphone) or (mobile phone) or (cellular phone)) and mobile device. The search string for outcome was: (2) ((weight loss) or (weight control) and (overweight) or (obesity)). The strings were combined into one large search string. Devices other than mobile phones were also searched separately and as part of the large search string. The Boolean search string for ‘other mobile devices’ was ((PDA) or (personal digital assistant)) or palmtop and (weight loss). The string was combined with the large string specified above and searched on CINHAL plus, Google Scholar, and the Cochrane library (for search details, refer to the appendix). The addition of the full string to the PubMed advanced search engine did not make the search significant, and the two strings were entered separately.

### 2.5. Inclusion and Exclusion Criteria

Systematic Review Inclusion Criteria:

(1) Randomized controlled trials on weight loss and mobile devices in overweight and obese adults without specified co-morbidities
(2) Weight as a primary or secondary outcome. Studies examining changes in diet and physical activity were included if weight was measured as a secondary outcome
(3) Studies published until May 2013
(4) New generation use mobile devices that are commercially available, including:
  ▪ Mobile phones and smartphones ( iPhones, Android phones, and Blackberries)
  ▪ Modern commercially available portable devices such as iPads, iPods, and MP3 players
  ▪ Personal Digital assistants (PDA’s). PDA’s were included if they were of newer generation with updated feedback thermometers installed to ensure relevance and modernity en par with newer generation devices.
(5) Studies measuring weight using validated weighing scales
(6) Studies published in the English language
(7) Open access peer reviewed journals
(8) Studies with pre and post intervention weight measures
(9) Clear description of intervention content including:
  ▪ Content of messages
  ▪ Techniques used

Systematic Review Exclusion Criteria:

(1) Case studies and quasi experimental studies
(2) Studies on diet or physical activity without weight either as a primary or secondary outcome measure
(3) Studies focusing on specific groups with pre-existing diseases and comorbidities in addition to overweight and obese patient status
(4) Studies in subjects <18 years of age
(5) Studies using Tele-monitoring devices alone such as weighing scales and accelerometers without additional mobile components such as a phone or PDA
(6) PDA’s without updated feedback thermometers installed
(7) Older generation handheld tablets not specified as PDA’s or any of the above listed devices, and not commercially available for wide population use
(8) Studies examining weight change by using the phone for phone calling purposes without employing mobile smartphone features
(9) Purely web-based interventions without a mobile device component
(10) Stationary electronic devices such as computers
(11) Laptops
(12) Studies published in languages other than English
(13) Studies employing subject self-report of weight change without objective validated measures
(14) Studies without a clear description of intervention content and techniques used

### 2.6. Data Extraction

Data were extracted for descriptive purposes. These included data on mean changes in weight and data on physical activity and dietary intake. Extracted data also included study characteristics such as study size, study design, methodology, participant demographics, theory, and intervention content and techniques. They were summarized in tabular format.

### 2.7. Study Quality Assessment

Trials were appraised using the Cochrane handbook for trial appraisal risk of bias, chapter 8 of the Cochrane handbook [28]. Trials with attrition of 47% and over, with significant differences in baseline characteristics were not included in the final analysis.

### 2.8. Data Coding

Extracted data on behavioural change techniques were coded according to the Michie and Abraham BCT coding criteria [15,16]. Extracted theoretical data were partially coded according to the Michie and Prestwich [27] theory coding criteria for items 1–5, item 15, and item 18 of the coding scheme. Items 7–11 were merged into one conceptual category.

### 2.9. Data Synthesis

Data extracted on mean weight loss measured in kg were pooled using Comprehensive Meta-Analysis Software version 2.0. Interventions reporting mean weight loss in LBS. were converted to kilograms. Percentage weight loss was converted to mean weight loss in kg by multiplying weight loss percentage by baseline weight and dividing this value by 100. The software calculated Cohen’s d for standardized differences in means. A random effects model was selected. The Cochrane handbook [29] recommends a random effects model when interventions are heterogeneous. Standard deviations for mean change in weight from baseline to follow-up were utilized in accordance with the handbook. Authors of studies not reporting differences in standard deviations for change were contacted. Standard deviations for weight change which were not directly reported were approximated from studies reporting indirect measures from which standard deviations could be calculated. Standard deviation was calculated from Cohen’s d by subtracting mean differences in weight loss between intervention and control groups and dividing this figure by the standardized difference in means. Standard Error for mean change was also approximated from studies reporting confidence intervals for mean weight loss by dividing the confidence interval (maximum-minimum) by the relevant t-distribution for sample size (N-1 degrees of freedom), multiplied by two. Standard deviation was obtained by multiplying the standard error by the square root of the sample size. Interventions with multiple control groups *versus* one intervention group were combined using guidelines in the handbook [29] by calculating pooled means, standard deviation, and overall N for both control groups in Microsoft excel.

### 2.10. Data Analysis

Tests for heterogeneity were undertaken using the Comprehensive Meta-Analysis software. Heterogeneity was assessed in accordance with the Cochrane handbook criteria for heterogeneity. The handbook categorizes moderate heterogeneity in accordance with I2 values between 30–60, high heterogeneity in accordance with I2 values > 60, and very high heterogeneity in accordance with I2 values between 75–100 [29]. Heterogeneity was assessed in accordance with these cut off criteria by the handbook in tandem with an evaluation of statistical significance, and the strength and direction of the overall effect as advised by the Cochrane handbook. Sensitivity analyses were also run to determine whether a single study provided a disproportionate contribution to the overall effect. Publication bias was assessed by generating a funnel plot to evaluate symmetry in the dispersion of effect estimates.

## 3. Results Part A: Systematic Review with Meta-Analysis

### 3.1. General Search Results

**Figure 1:**
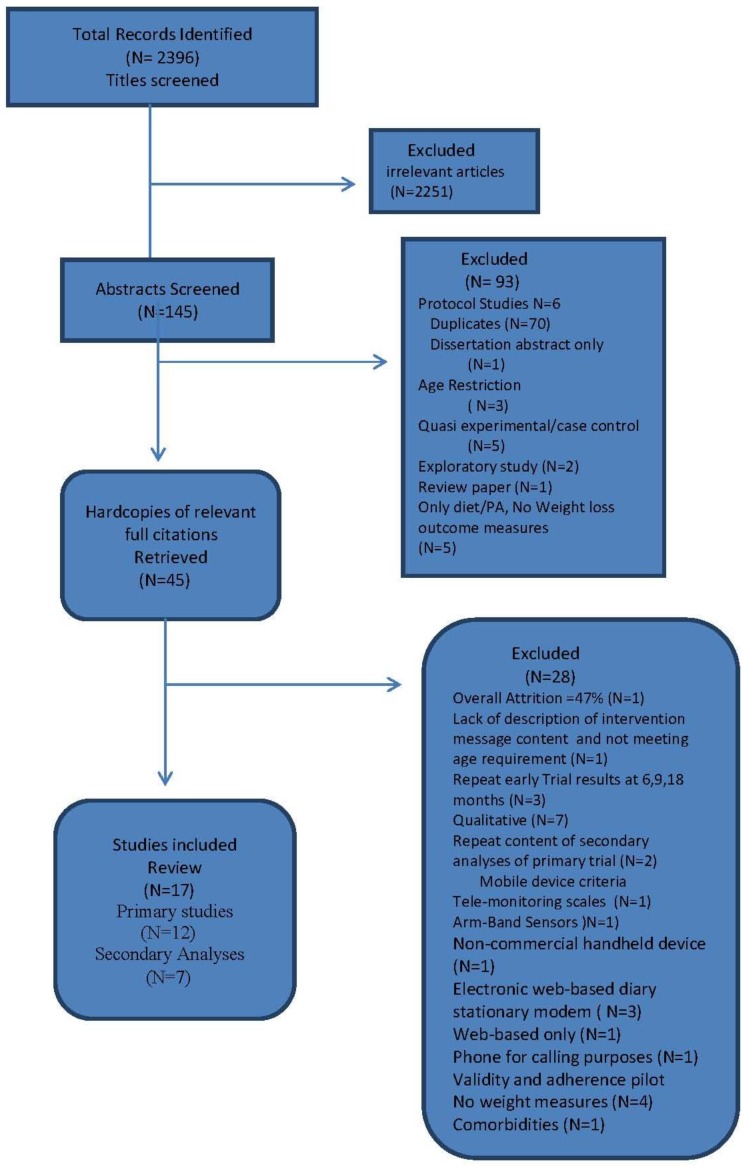
Flow Chart of Search.

The search generated 2396 studies. After title screening for relevance to the research question, 145 abstracts were read. Of these, 70 were duplicates and were removed. Another 23 were removed as they did not meet inclusion criteria. The reasons for not meeting inclusion criteria are detailed in the flow chart (Figure 1) and included: protocol studies without published results, electronic full text unavailable, not meeting age restriction of subjects, lack of weight loss measures, and methodology. A total of 45 articles were read in full. Of these, a total of 24 met inclusion criteria and were selected for review. The other 21 studies were excluded because they were repeats of secondary analyses with similar information, did not provide pre and post weight loss measures, or did not meet mobile device requirements (*i.e.*, tele-monitoring scales, purely web-based, or utilized phones for calling purposes). Studies which did not provide sufficient information on mobile intervention content and techniques utilized were not included. Additionally, studies which had consecutive findings from lengthy trials at several early intervals were excluded since the final published results were selected. A total of 17 studies were selected for review. A summary of the CINAHAL search is found in the Appendix Table A1.

### 3.2. General Descriptive

A total of 17 randomized controlled mobile device weight loss trials were included in the analysis, summarized in Table 1. Of these, 12 RCTs were primary studies [30,31,32,33,34,35,36,37,38,39,40,41]. The remaining five studies were secondary analyses of the primary studies [42,43,44,45,46]. Eight studies were two group RCTs including Haapala [30], Hurling [31], Patrick [32], Shapiro [33], Turner-Mcgrievy [34,35], Brindall [36], and Spring [37]. Studies [30,31,32,33,34,35,36,37,38] were two group RCTs. The remaining four studies were all three arm parallel RCT’s including studies by Carter [38], Prestwich [39], Napolitano [40] and Burke [41]. The most common form of subject recruitment included newspaper ads or newsletters [30,32,34,35,36,38,40], emails [34,35,38,40], and online advertising [32,33,34,35,38,40]. One study used a commercial recruitment agency [31] and another study utilized Facebook [40] as a means of subject recruitment. Sample size varied from 52 subjects [40] to 210 subjects [41]. Mean subject age ranged from 20.4 years [33] to 57 years of age [30]. Trial length ranged from 4 weeks [32] to 2 years [30]. Mean BMI ranged from 26 kg/m^2^ [30,31] to 34 kg/m^2^ [36,38]. Two studies did not report mean subject BMI. Study locations included the UK, USA, Finland, and Australia. A total of seven studies were undertaken in the USA [37,41], three studies in the UK [31,38,39], one study in Finland [30], and one study was undertaken in Australia [36]. Most of the studies consisted of primarily female subjects ranging from 64% [39] to 100% [36] of the sample. Only one study had a predominance of male subjects, with 84% of the sample being male [37]. In addition to this, one study had a roughly equal representation of male and female subjects [33].

### 3.3. Mobile Device Intervention Media

A total of 8 out of the 12 interventions had a mobile phone as an intervention medium [30,31,32,33,36,38,39,40]. Three studies did not utilize a mobile phone component [34,35,41]. Two of these studies utilized a podcasting component employing an Mp3 player or an iPod as an intervention medium [34,35]. The remaining two studies used a PDA for weight loss [37,41], of which one study combined a PDA with a mobile phone for calling purposes only [37].

### 3.4. Target Behaviour and Weight Loss

Nine studies targeted both diet and physical activity to induce weight loss. The remaining three studies concentrated primarily on physical activity to induce weight loss [31,33,39]. Four studies had pre-determined behavioural goals set for all subjects [30,32,39,40], while subjects determined their own goals in the remaining studies. Goals ranged from specific caloric reduction goals [30,32,40] to physical activity goals [39,40]. The Patrick *et al.* study had a 500 daily caloric reduction goal set for all participants [32]. The Prestwich *et al.* study had physical activity goals of 30 min of walking for 5 days a week, with a minimum of 10 min bouts of vigorous physical activity [39]. Additionally, the Napolitano *et al.* study had a physical activity goal of 250 min per week for all participants [40]. Two studies set caloric reduction goals or modified goals in accordance with a given subject’s weight [37,40]. In most studies, subjects set their own weight loss goals. Three studies had pre-set weight loss goals for study participants. The Haapala *et al.* study [30] had a pre-set weight loss goal for participants at 2 kg/month. The study by Napolitano *et al.* [40] had a weight loss goal of 2 pounds per month for all participants. The Spring *et al.* study [37] had a weight loss goal of 5%–10% in accordance with individual body weight.

**Table 1:** Study Characteristics of Mobile Phone Interventions.

Study	Location	Sample	Trial Length	Design/ Recruitment	Target Behaviour	Experimental Group	Control Group	Measures	Results
Haapala *et al.* 2009 [30]	Finland	N = 125 F = 78% M = 21% Mean BMI = 26.3 kg/m^2^ Range = 19–30 kg/m^2^ Mean Age = 38 Range = 25–44 women = 80% White = 75%	12 months	Two Group RCT Newspaper, Phone screening	Diet, PA	Mobile phone programme Weight Balance Calculates PA/Energy Expenditure + Tailored Daily Target Goal Text Messages, Time left for target reach Weight loss targeted at 2 kg/month	No Intervention	Self- administered questionnaires on Diet + PA + Monthly weight recorded by Nurse Waist circumference measures in cm via tape	Weight Loss EG = 4.5 kg over 12 months *p-*value *vs.* CG = 1.1 kg over 12 months (*p*-value for group differences = 0.006)
									Weight% lost EG = 5.4% *vs.* CG = 1.3% (*p*-value < 0.006) Waist Circumference EG = 0.6 reduction from baseline (SD = 1.7) CG = 0.4 reduction (SD = 6.6)
									Secondary Outcomes Self-Efficacy Score EG = 0.6 increase from baseline CG = 0.4 increase from baseline ED score EG = 0.4 (SD = 0.06) reduction from baseline CG = 0.1(0.7)
Hurling *et al.* 2007 [31]	Bedfordshire UK	N = 77 EG = 47 CG = 30 Mean Age = 40.4 Range = 30–55 Mean BMI = 26 kg/m^2^ Range = 19–30 kg/m^2^	9 Weeks	Two Group RCT Randomization = Random Stratification Market Research Agency Recruitment + Phone Screening	PA	Mobile phone and internet Weekly PA sessions with reminders Via phone /email Automated Feedback on PA levels + tailored solutions texts for barriers	No Intervention	Wrist Accelerometer Bluetooth Actiwatch + Self-Report of PA Weight- Bio-Electrical Impedance Scales	Primary Outcomes- EG = increase over baseline perceived control (*p*-value < 0.001) + Intent to exercise (*p*-value < 0.001 PA levels EG = increase in moderate PA (*p*-value = 0.03) Mean increase PA relative to control = 2 h 18 min per week Secondary Outcome- Weight Loss EG = 2.18% body fat(SD = 0.59) CG = 0.17% body fat loss (SD = 0.81) (*p*-value = 0.04)
Patrick *et al.* 2009 [32]	San Diego California USA	N = 93 EG = 65 CG = 33 Mean Age = 45 Range = 25–55 Mean BMI = 32.2 kg/m^2^ Range = 25–39.9 kg/m^2^ F = 80% M = 20%	Four Months	Two Group RCT Simple Randomization Recruitment via Flyers, newspapers, adds, announcement on Craigslist	PA, Diet	Printed Monthly Materials Weight Control Brief Monthly Phone Calls from Counsellor 5–15 min Tailored and Interactive SMS + MMS Frequency = 2–5/day ½ messages requested reply Users select frequency of texts/time of day Text Topics- Goal setting, volumetric, meal planning, eating out, Healthy food environment Goals-500 Calorie Reduction/Daily	Printed Monthly materials on weight control	Weight measured via calibrated weight scales in study offices + Self-report of weight 1×/week via mobile phone	Primary Outcome-Weight EG four month weight loss *versus* control group difference = (lost) −1.97 kg (95% CI = −0.34 to −3.60 kg) (*p*-value = 0.02)
									Adjusted for Age + Sex EG *versus* CG weigh loss = 2.88 kg, total 3.16% difference in weight loss
									Secondary Outcome-Satisfaction −92% would recommend intervention
Norman *et al.* 2013 [43]	San Diego California USA	N = 93 EG = 65 CG = 33 Mean Age = 45 Range = 25–55 Mean BMI =32.2 kg/m^2^ Range =25–39.9 kg/m^2^ F = 80% M = 20%	Four Months	Secondary Analysis of above study; Patrick *et al.* 2009 [32] Two Group RCT Simple Randomization Recruitment via Flyers, newspapers, adds, announcement on Craigslist	Nutrition, Fruit + Vegetable Intake, Eating Behaviour Change	Printed Monthly Materials Weight Control Brief Monthly Phone Calls from Counsellor 5–15 min Tailored and Interactive SMS + MMS	Printed Monthly materials on weight control	Nutrition intake via multiple 24 h food recall Eating Behaviour Inventory Score Changes (EBI)	EG baseline EBI score = 70.88( SD = 6.2) 4 month after = 79.62 (8.11) Total Change = 8.73 (SD = 6.23) (*p*-value ≤ 0.001) EG Fruit + Veg intake Baseline score = 4.60 (SD = 3.01) 4 months after = 5.08 (SD = 3.48) Total Change = 0.49 (2.33) (*p*-value = 0.297) CG baseline EBI score = 72.19 (SD = 7.57) 4 months after = 74.23 (SD = 6.58) Total Change = 2.04 (SD = 0.02) (*p*-value = 0.140) Baseline Fruit Veg intake = 5.84 (SD = 3.04) 4 months after = 4.33 (SD = 2.69) Total Change = −1.52 reduction (SD = 4.22) (*p*-value = 0.079)
Carter *et al.* 2012 [38]	Leeds UK	N = 128 Age-18–65 Mean age = 41 F = 68.8% Mean BMI = 34 kg/m^2^ (SD = 5) F = 77% M = 23%	6 months	3 Arm Parallel RCT Random process of Minimization Recruitment- Email Newsletter, Internet, Posters, from Large Local Employer	PA + Diet	Smartphone App My Meal Mate Diary App stores food photos incorporates Goal Setting, Self-Monitoring, and feedback Via weekly mobile text messages	Either Website or Diary (without App and mobile components)	Portable Weight Scales Weight Watchers 89584 Model	Primary Outcome Adherence EG = increased adherence relative to control 92 days (SD = 67) *vs.* control 35 days (SD44) Secondary Outcome Weight BMI kg/m^2^ smartphone EG group reduction = −1.6 kg/m^2^ reduction (95% CI = −2.2–1.1) CG Web group = BMI reduction −0.5 kg/m^2^ (95% CI = −0.9–0.0) CG Diary Group = BMI reduction −1.0 kg/m^2^ (95% C = −1.6–0.4) Body fat EG Smartphone % reduction = −1.3% reduction (95% CI = −1.7–0.8) CG Diary Group = % body fat reduction −0.09% (95% CI = −1.5–0.4) CG Website group = −0.5% reduction Body fat (95% CI =−0.90–0) Mean Weight Change (from baseline) EG smartphone reduction = −4.6 kg (95% CI = −6.2–3.0) CG Diary Group = Mean weight change reduction = −2.9 kg (95% CI = −4.7–1.1) CG Website Group = Mean Weight reduction = −1.3(−2.7–0.1)
**Study**	**Location**	**Sample**	**Trial Length**	**Recruitment**	**Target Behaviour**	**Intervention**	**Controls**	**Measures**	**Outcome**
Shapiro *et al.* 2012 [33]	San Diego California	N = 170 F = 67 M = 64 Mean Age = 41.9 Range = 25–69 Mean BMI = 32.2 kg/m^2^ Range = 25–39.9 kg/m^2^	12 months	2 Group RCT Recruitment- Magazine, online advertising	Primarily PA, diet secondary	Mobile phone SMS + MMS personalized + interactive Frequency 4× day/12 months with feedback (graphical step feedback) Messages tailored based on online baseline survey Message content— self-monitoring PA, diet, sugar sweetened beverages, Knowledge questions, tips, educational facts, portion control	Monthly newsletters	Yamax Digi-Walker CW Series 600 Pedometer Weight measures -Digital Weight Scale	No group differences in weight loss at 6 months CG weight reduction = 1.53 lb. EG weight reduction 3.72 lb. 12 months CG weight reduction 2.27 lb *vs.* 3.64 lb. EG Adherence = 60%–69%
									Increased adherence = greater weight loss at 6 months (*p*-value- = 0.039 and 12 months (*p*-value = 0.023)
									EG group step counts increased to 3000 step/day (*p*-value < 0.05)
									Increased step count = increased weight loss (*p*-value < 0.05)
**Study**	**Location**	**Sample**	**Trial Duration**	**Recruitment**	**Target Behaviour **	**Intervention**	**Control**	**Measures**	**Outcome**
Turner-Mcgrievy *et al.* 2011 [34]	Raleigh Durham, North Carolina USA	N = 96 EG = 47 CG = 49 Age Range = 18–60 Mean Age = 38 Mean BMI = 32.6 kg/m^2^ Range 25–45 kg/m^2^ 73% = female 78% = White	6 months	2 Group RCT Computerized random number generator randomization Recruitment- T.V advertisement, email	PA + diet	Podcast on Diet + PA information, goal setting, soap opera, audio blog of a man/woman losing weight founded on earlier study using social cognitive theory + Mobile component with PA App (iPhone, iPod, Blackberry), interacting on Twitter with study participants and counsellors Frequency = 2 podcasts per week for three months 15 min each + 2 mini podcasts per week for 3–6 months 5 min each Study coordinator sent 2 messages a day to study group	Podcast only on diet + PA designed on earlier study using social cognitive theory Received handbook on calorie content of food Podcast frequency 2 podcasts per week for three months 15 min each with 2 mini podcasts per week for 3–6 months 5 min each	Body Weight scale Fat Secret Calorie Counter Scale	Weight Loss did not differ by groups (*p*-value = 0.98) Mean weight loss % EG = −2.7% (SD = 5.6) CG = −2.7% (SD = 5.1) EG 3× more likely than CG to use app to self-monitor diet (*p*-value = 0.01) Number of podcasts download correlated with weight loss Pearson’s r = −0.46 (*p*-value = 0.001) EG has increased sense of self control at 3 months *vs.* controls (*p*-value = 0.02), but not at 6 months (*p*-value = 0.06) CG relied more on friends for support (*p*-value = 0.045) 28% *vs.* EG relied on social groups online for support (*p*-value = 0.001) 25% EG *vs.* 0% CG
*Turner-Mcgrievy 2013 [44] Secondary analysis of Turner-Mcgrievy 2011 study [34]	Raleigh Durham area North Carolina USA	N = 96 BMI = 25–45 kg/m^2^ Age 18–60 Mean = 38	6 months	Post-Hoc Secondary Analysis of RCT (Mcgrievy *et al.* 2011 [34]) Recruited via email, televisions adds, and newspapers	PA	PA App With podcasts	No App Only podcast Paper Journal and Website Controls	Body Weight scale Fat Secret Calorie Counter Scale	EG self-monitored more frequently relative to CG = 2.6 (SD 0.5) days/week *vs.* 1.2 (0.5) days/week CG (*p*-value < 0.001) EG had increased intentional PA relative to CG = 196.4 (SD = 45.9) kcal/day *vs.* 100.9 (SD = 45.1) (*p*-value = 0.02) BMI EG lower at 6 months relative to controls = 31.5 (SD = 0.5) kg/m^2^, CG = 32.5 (0.5) kg/m^2^ (*p*-value = 0.02) No group difference in frequency of self-monitoring (*p*-value = 0.63) EG consumed less energy relative to controls 1437 (SD = 188) kcal/day *vs.* CG paper journal 2049 (SD = 175) kcal/day (*p*-value = 0.01)
Turner-Mcgrievy 2009 [35]	Raleigh Durham Area North Carolina	N = 78 BMI = 25–45 kg/m^2^ Mean age EG = 37.7 Mean Age CG = 39.6 Female = 80% White = 71%	12 weeks	2 Group RCT Recruitment via newspapers + University email	PA, Diet	24 enhanced podcast episodes designed on social cognitive theory Frequency 2 podcasts per week Mean Length 15 min 42 s Delivery via MP3 player Content targeted 5 areas of social cognitive theory – expectancies, expectation, self-efficacy behavioural capability Using health education on nutrients/PA + soap opera podcast, information of benefits of weight loss, podcast discussing expectations during weight loss+ strategies and end of podcast goal setting with self-monitoring Groups given book on calorie content of food items	24 standard podcast episodes based on commercial weight loss program Frequency 2 podcasts per week Mean Length18 min 34 s Delivery via mp3 player Content used cognitive restructuring to avoid over-eating focused on how to lose weight conducted by 2 hosts Including stimulus control to avoid snacking and positive psychology to improve body image Groups given book on calorie content of food items	Body weight scale measured in study office baseline + follow-up Self-report PA Nutritional intake assessed using Prime Screen Questionnaire, averaging intake fruit, vegetables, and fat Likert scale used to assess level of control + elaboration	Enhanced podcast Group Weight loss −2.9 kg (SD = 3.5)* vs.* Control standard podcast = −0.3 (SD = 2.1) BMI change Enhanced Podcast Group = −1.0 kg/m^2^ (SD = 1.2); Control standard podcast group = −0.1 (0.7) kg/m^2^ Between group difference *p*-value ≤ 0.001 Enhanced podcast group Fruit and vegetable intake increase = 0.4 (SD = 0.7) fruit 0.2 (SD = 0.9) vegetable intake Control standard podcast fruit+ veg intake increase = 0.01 (SD = 0.4) fruitDecrease vegetable intake of = −0.2 (SD = 0.7) *p*-value between group differences ≤ 0.005 Increase in reported vigorous activity enhanced podcast group = 0.8 (SD = 0.9) days per week *vs.* control decrease of vigorous activity = −0.4 (SD = 1.4) *p*-value between groups ≤ 0.01 No difference in high fat food intake between groups Increase in knowledge scores for enhanced podcast group
**Study**	**Location**	**Sample**	**Trial Length**	**Design/ Recruitment**	**Target Behaviour**	**Intervention**	**Control**	**Measures**	**Outcome**
Prestwich *et al.* 2010 [39]	United Kingdom	N = 149 Mean age = 24.44 F = 64% M = 36% BMI N/A	4 weeks	3 Group RCT Recruitment via email Allocation sequence based on computer generation randomization used (no stratification or block methods)	PA	Group 1 = Implementation and intentions + SMS plan Tailored text messages reminding to initiate plan according to participant determined scheduling Required to plan 30 min 5 days a week of walking(at least bouts of 10 min brisk walking) in specific achievable environments/situations Frequency = 1 text per plan, scheduled at same time of plan behaviour Group 2 = Implementation + SMS goal Same requirements as group 1 But did not receive plan reminder text, instead goal reminder of brisk walking time of texts individually tailored by determining timing All groups Provided with Government recommended guidelines of 30 min moderate to vigorous physical activity and information on brisk walking for 30/min day on 5 or more days/week End of task given plan recall + goal recall task	Control Group 3 did not receive any text messages + no requirement to form implementation intentions Goal recall task end of study	Self-Reported PA levels Physiological measures taken BMI at study site using digital scale at baseline + follow-up	Primary outcome- physical activity 42% in the Intentions + goal reminder group increased brisk walking for 2 more days a week 45% in the Intentions+ plan group increased brisk walking for 2 more days/week relative to 22% of controls *p* values ≤ 0.01 Secondary outcomes Weight loss: Implementation Intention+ goal reminder lost 0.53 kg *Vs.* Implementation Intention+ plan group = 0.10 kg (*p*-value group difference = 0.03 95% CI = 0.04–0.91), when comparing intention+ goal group with intention+ plan and control effect remains significant = *p*-value 0.046 (95% 0.03–0.72) Control group = 0.14 kg Impact on other PAIntention +plan group increased other types of activity *vs.* control (*p*-value < 0.03) but not relative to the other group (*p*-value 0.12) Plan recall higher in intention +plan group relative to intention +goal group (*p*-value < 0.01)
**Study**	**Location**	**Sample**	**Trial Length**	**Recruitment/ Randomization**	**Target Behaviour**	**Intervention**	**Control**	**Measures**	**Outcome**
Brindall *et al.* 2013 [36]	Australia	N = 53 BMI = 26–34 kg/m^2^ Mean BMI = 34 kg/m^2^ 100% female Age 19–63 Mean age = 42	8 weeks	2 group RCT randomization using a computer generated sequence Recruitment = Newspaper add and established volunteer database	Diet+ PA	iPhone required commercially available partial meal replacement programme Intervention group given Meal replacement App Instructed on MRPP celebrity slim app App replaces meals 2× a day with shakes (does not count calories, only restricts energy intake) Intervention support app with the following over control app: Rewards positive behavioural change prompts self-monitoring with reminders + tailored feedback	iPhone required commercially available partial meal replacement programme Control given Static App based on info in the MRP App Instructed on Meal replacement (MRP) app celebrity slim App replaces meals 2× a day with formulated shakes does not count calories, only restricts energy intake	Self-report of weight In person weight recording at study office with body weight scale + stadiometer for height	Mean difference in weight loss between EG + CG was not significant EG mean weight loss difference = 3.2% (SD = 0.38) CG mean weight loss difference = 2.2% (SD = 0.37) (*p*-value = 0.08) Week 8 = 23% CG and 21% EG lost 5% body weight 95% support app found it helpful with maintaining scheduled goals *vs.* 9% in standard app group Mean increase in positive affect in group with support app = 0.48 increase (SD = 0.14) * vs.* decrease in the standard app CG = −0.01 (SD = 0.13)
						Contains trophy room for rewards, goals/tasks to finish, Meal calendar, weight recorder + health information Prompting generated using Apple Push Notification Service frequency 3 × day during meal times and leisure PA times Individually tailored to schedule Meal Calendar on iPhone used for self-monitoring and caloric/energy expenditure feedback Message board on iPhone provides motivational messages			
**Study**	**Location**	**Sample**	**Trial Length**	**Recruitment**	**Target Behaviour**	**Intervention**	**Control**	**Measures**	**Results**
Napolitano *et al.* 2013 [40]	Eastern United States (large urban University	N = 52 students Age = 18–29 Mean age = 20.47 BMI = 25–50 kg/m^2^ Mean BMI = 31.36 kg/m^2^ Female = 86.5%	8 weeks	3 arm RCT (pilot) Recruitment = Emails, listervs, online newspapers, flyers, Facebook, university student organizations	Diet +PA	Group 1 = Facebook only Weekly hand outs and podcasts /videos on health education topics( planning +nutrition, PA, hunger triggers, social support, dinning out, relapse prevention) Physical activity and eating healthy event invitations Targets gradually increase PA to 250 min/week Caloric intake 1200–1800 kcal/week according to weight Group 2 = Facebook + mobile phone text messaging Received same components as group 1 except belonged to a different Facebook group and text messages on goal setting, self-monitoring + social support, positive reinforcement + brief feedback	Control group wailing list	Body weight measured using calibrated weight scale at 4 and 8 weeks + height measured via stadiometer Calorie Counter, Pedometer, Digital scale for in person recording	8 weeks weight loss Facebook Plus texting group = −2.5 kg (SD = 2.4)Facebook group =−0.63 (SD = 2.4) Waiting list Control = −0.24 (SD = 2.6) Changes stat sig between groups (*p*-value = 0.05) Program helpfulness = 97% agree 100% recommend programme 81.3% found videos + hand outs helpful
**Study**	**Location**	**Sample**	**Trial Length**	**Recruitment**	**Target Behaviour**	**Experimental Group**	**Control Group**	**Measures**	**Results**
						Messages topics differed each day 3 types—self monitoring, prompting to self-monitor, and texts tailored according to individual barriers Personalized feedback provided via summary reports Contained Buddy component for peer support Given a pedometer, calorie counter book, and digital scale Weight goals = max 2 pounds loss/month set by staff			
**Study**	**Location**	**Sample**	**Trial Length**	**Design**	**Target Behaviour**	**Experimental Group**	**Control Group**	**Measures**	**Results**
Spring *et al.* 2013 [37]	Midwestern VA Hospital USA	N = 69 Mean Age = 57 85% Male	12 months	2 group RCT Recruitment by contacting all outpatients	Diet + PA	EG received PDA to monitor diet+ PA with thermometer providing automated feedback With mobile phone coaching calls for 6 months 10–15 min advice, providing tailored timely feedback Attended same Move sessions as controls Calorie goals tailored to baseline weight +activity goals 5%–10% weight loss goal	Bi-weekly weight loss groups (Move sessions) in person VA outpatient clinic Duration = 1.5 h led by psychologists, nutritionists, and dieticians	Calibrated weight scale used to measure weight at study sites	EG 3/9 kg (3.1%) more weight loss relative to control (95% CI 2.2–5.5) No evidence treatment varied across time (*p*-value = 0.44) OR 5% weight loss EG to CG = 6.46 (95% CI 2.5–18.6) With no variation across time (*p*-value = 0.13)
**Study**	**Location**	**Sample**	**Trial Length**	**Design**	**Targets** **Behaviour**	**Experimental Group**	**Control Group**	**Data Collection**	**Results**
Burke *et al.* [41,42] 2011; 2012 (repeat analyses of SMAR TRIAL)	Pittsburgh Pennsylvania USA	N = 210 Mean age = 46.8 Mean BMI = 27 kg/m^2^ 84% female	24 months	SMART Trial 3 group RCT	Diet+ PA	Group 1 = PDA+ Feedback Group 2 = PDA only All groups had weekly groups sessions 1–4 months, bi-weekly months 5- components goal setting self-monitoring dietary intake + nutritional goals, weekly exercise goalsdietary goals 1200–1800 Calories per day, with no more than 25% calories from fat Increase PA to 180 min over 6 months, with 30 min increases in concurrent months	Group 3 control = differed in self-monitoring method-paper diary All groups had weekly groups sessions 1–4 months, bi-weekly months 5–12 components goal setting self-monitoring dietary intake + nutritional goals, weekly exercise goals dietary goals 1200–1800 Calories per day, with no more than 25% calories from fat Increase PA to 180 min over 6 months, with 30 min increases in concurrent months	Digital scale to measure weight by study staff Self-reported PA 2 unannounced 24 h dietary recalls	Waist circumference decreased more in PDA groups relative to paper group (*p*-value = 0.02) Energy + saturated fat intake decreased in PDA groups relative to paper group (*p*-value = 0.05) Only PDA +FB lost significant weight = –2.32(95% CI = −4.29–0.35) (*p*-value = 0.02) Paper Group = −1.94 (95% CI = −3.88–0.01) PDA Group = −1.38 (95% CI = −3.38–0.62 Increased weight loss for more adherent >60% *vs.* less adherent <30 (*p*-value < 0.001)
**Study**	**Location**	**Sample**	**Trial Length**	**Design**	**Targets**	**Experimental Group**	**Control Group**	**Measures**	**Outcome**
Archaya *et al.* 2011 [45] Secondary Analysis of Burke *et al.* [41,42]	Pittsburgh Pennsylvania USA	N = 210 Mean age = 46.8 Mean BMI = 27.4 kg/m^2^ 84% female	24 months	SMART Trial 3 group RCT Secondary analysis	Diet+ PA	Group 1 = PDA+ Feedback Group 2 = PDA only All groups had weekly groups sessions 1-4 months, bi-weekly months 5- components goal setting self-monitoring dietary intake + nutritional goals, weekly exercise goals dietary goals 1200-1800 Calories per day, with no more than 25% calories from fat Increase PA to 180 min over 6 months, with 30 min increases in concurrent months	Group 3 control = differed in self-monitoring method-paper diary All groups had weekly groups sessions 1-4 months, bi-weekly months 5-12 components goal setting self-monitoring dietary intake + nutritional goals, weekly exercise goals dietary goals 1200-1800 Calories per day, with no more than 25% calories from fat Increase PA to 180 min over 6 months, with 30 min increases in concurrent months	Digital scale to measure weight by study staff Self-reported PA 2 unannounced 24 h dietary recalls	PDA groups increased fruit consumption relative to controls (*p*-value = 0.02) and vegetable consumption relative to controls (*p*-value < 0.01) Frequent self-monitoring associated with total sugar (*p*-value = 0.02) in both groups Interaction between self-monitoring in both PDA groups and changes in fat intake (*p*-value = 0.02), trans-fatty acids (*p*-value = 0.04), mono saturated fats (*p*-value = 0.002)
**Study**	**Location**	**Sample**	**Trial Duration**	**Design**	**Targets**	**Experimental Group**	**Control Group**	**Measures**	**Outcome**
Conroy *et al.* 2011 [46] Secondary analysis of Burke *et al.* 2011 [41,42]	Pittsburgh Pennsylvania USA	N = 210 Mean age = 46.8 Mean BMI = 27.4 kg/m^2^ 84% female	24 months	SMART Trial 3 group RCT	Diet+ PA	Group 1 = PDA+ Feedback Group 2 = PDA only All groups had weekly groups sessions 1–4 months, bi-weekly months 5- components goal setting self-monitoring dietary intake + nutritional goals, weekly exercise goals dietary goals 1200–1800 Calories per day, with no more than 25% calories from fat Increase PA to 180 min over 6 months, with 30 min increases in concurrent months	Group 3 control = differed in self-monitoring method-paper diary All groups had weekly groups sessions 1–4 months, bi-weekly months 5–12 components goal setting self-monitoring dietary intake + nutritional goals, weekly exercise goals dietary goals 1200–1800 Calories per day, with no more than 25% calories from fat Increase PA to 180 min over 6 months, with 30 min increases in concurrent months	Digital scale to measure weight by study staff Self-reported PA 2 unannounced 24 h dietary recalls	PDA_+FB mean number of self-monitoring entries greater than PR arm (3.4* vs.* 2.4) (*p*-value = 0.003) More likely to maintain high adherence to PA goals overtime than PDA (*p*-value = 0.02) or PR arm (*p*-value = 0.0003)

EG = Experimental Group; CG = Control Group; PA = Physical Activity; 95% CI = 95% Confidence Interval; OR = Odds Ratio; PDA = Personal Digital Assistant; SMS = Short Message Service FB = feedback.

### 3.5. Dietary Measures

Diet was most often measured using subject self-report of dietary intake compared to baseline reports. The most common dietary variables measured were changes in fruit and vegetable intake compared to baseline intake levels in the intervention and control groups. Fruit and vegetable intake calculations were different between the studies. Study [35] used the Prime Screen Questionnaire to assess fruit and vegetable intake, with scores of 0 indicating consumption frequency of less than once a week, scores of 1 indicating a consumption frequency of once a week, scores of 2 indicating consumption frequency of 2–4 times per week, scores of 3 indicating daily consumption, and scores of 4 indicating daily consumption at a frequency of twice per day. Study [43] calculated fruit and vegetable intake per 1000 kcals as a mean value of two 24 h food frequency recalls as well as study [45]. Other dietary variables of interest across the studies included changes in sugar intake and total fat, including, mono saturated, and trans-fatty acid intake. Dietary changes were also assessed in accordance with daily caloric intake relative to baseline caloric intake measured in kcal/day of energy consumption. Changes in dietary behaviour were measured using different questionnaire scores ranging from the eating density score utilized in one study [30] to the eating behaviour inventory score utilized in another [43]. Decreases in the ED score indicated favourable outcomes, demonstrating reduced energy dense caloric intake [30]. Increases in the EBI score which is measured on a 5 point scale with scores from 26–30 indicated positive changes, demonstrating behavioural change favouring healthy food intake [43]. None of the studies utilized objective markers of dietary intake by measuring nutrition biomarkers in serum samples of subjects.

### 3.6. Dietary Changes Overview

Most of interventions measuring changes in dietary intake and dietary behaviour found that subjects in the intervention groups had improvements in several indicators associated with improved dietary intake.

#### 3.6.1. Dietary Changes in Fruit and Vegetable Intake

Three studies measured changes in fruit and vegetable intake [35,42,43]. Changes in fruit and vegetable intake were favourable across the studies which measured changes in diet as a primary or secondary outcome. Study [35] found that intervention subjects in the enhanced podcast group had increased their levels of daily fruit intake compared to baseline levels by 0.4 points (SD = 0.7) and vegetable intake by 0.2 points (SD = 0.9) on the Prime Screen Questionnaire. By contrast, controls in the standard podcast group increased their fruit intake by a smaller amount, with score increases of 0.01 points (SD = 0.4). Controls also decreased their vegetable intake from baseline by 0.2 points (SD = 0.7). The differences between the intervention and control groups were significant (*p*-value < 0.005). In a secondary analysis of the Patrick *et al.* study, Norman *et al.* [43] found that the intervention arm receiving tailored daily SMS and MMS had increased their fruit and vegetable intake relative to baseline levels by 0.49 points (SD = 3.48). However, this was not significant (*p*-value = 0.297). They also had higher levels of fruit and vegetable intake compared to controls receiving monthly health newsletters who decreased their total fruit and vegetable intake compared to baseline measures by −1.52 points (SD = 4.22), but this was weakly not significant (*p*-value = 0.079) [46]. The secondary analysis of the SMART trial [42] found that the PDA intervention arms increased their fruit consumption compared to the control group utilizing paper methods for self-monitoring dietary intake (*p*-value = 0.02). They also increased their vegetable consumption relative to controls (*p*-value < 0.01).

#### 3.6.2. Dietary Changes in Sugar and Fat Intake

The SMART Trial specifically measured changes in sugar and fat intake [42,45]. The trial found that the experimental arms had decreased their saturated fat intake relative to the control group assigned to a paper self-monitoring method of dietary intake. Differences in saturated fat intake were marginally significant between the two experimental PDA arms relative to the control arm (*p*-value = 0.05). However, one study did not find an association between changes in total fat intake between groups receiving a standard podcast compared to an enhanced podcast [35]. The secondary analysis of the Burke *et al.* study [45] found that there was an interaction between self-monitoring and changes in total fat, mono saturated fat intake, and trans fatty acid intake in both of the PDA groups. Higher self-monitoring adherence resulted in reduced intake of these fats (*p*-value = 0.02). The same interaction relationship between the variable sugar intake and self-monitoring was observed in the experimental PDA arms (*p*-value = 0.002) [45].

#### 3.6.3. Dietary Changes in Daily Caloric Intake

Two studies measured changes in daily caloric intake. The Turner-Mcgrievy *et al.* (2013) [44] post hoc analysis of the 2009 study found that the experimental arm consumed less calories/day at 1437 kcal/day (SD = 188) relative to controls consuming 2049 kcal/day (SD = 175). The differences between groups were significant (*p*-value = 0.01). However, the Haapala *et al.* study [30] did not find significant differences in energy intake changes measured in kJ/day in the experimental group receiving a mobile phone intervention relative to the control group.

#### 3.6.4. Changes in EBI and ED Scores

Two studies measured changes in energy density and eating behaviour inventory scores. Both studies had results favouring the mobile device intervention groups [30,43]. The Haapala *et al.* [30] study found that subjects in the mobile phone intervention had reduced their energy dense (ED) score from baseline scores by 0.4 points (SD = 0.06), indicating positive changes in daily energy dense food consumption (*p*-value < 0.001) at all-time points of the 12 month intervention. The control group had a smaller reduction in their eating density score by 0.1 points (SD = 0.7) which was non-significant (*p*-value > 0.05). The differences between the intervention and control groups were significant at 12 months (*p*-value = 0.003). The secondary analysis of the Patrick *et al.* study [43] found that the experimental group receiving SMS and MMS had positive changes in their eating behaviour inventory score, suggesting favourable changes in dietary intake with an improvement in the score by 8.73 points from baseline (SD = 6.23) (*p*-value ≤ 0.001) after the four month intervention. The control group by contrast, had smaller positive changes in their dietary intake, with a total increase of 2.04 points (SD = 6.58) over their score at baseline (*p*-value = 0.140) at the end of the four month study [43].

### 3.7. Physical Activity Measures

Physical activity levels were measured using different methods ranging from self-report of physical activity to objective accelerometer or pedometer physical activity data with graphical MMS feedback charts. Physical activity was expressed as increases in hours or days of physical activity per week, changes in energy expenditure measured in kcal/day over baseline levels, and changes in steps per day [31,33,34,35,39].

### 3.8. Physical Activity Overview

The interventions measuring physical activity levels as a primary or secondary outcome mostly found that physical activity levels increased in the mobile device intervention groups relative to the control groups [31,33,34,35,39]. Indirect measures of physical activity were also reported across the studies, with increased adherence to physical activity goals [31,39].

#### 3.8.1. Perceived Physical Activity Goal Adherence

In a secondary analysis of the SMART study, study [46] found that the intervention group assigned to the PDA plus feedback group had higher levels of adherence to physical activity goals relative to the PDA only group (*p*-value = 0.02) and the paper self-monitoring group (*p-*value = 0.0003).

#### 3.8.2. Changes in Physical Activity Levels

A total of five studies which measured changes in physical activity levels had results favouring the mobile device the intervention groups [31,33,34,35,39]. The Hurling *et al.* study [31] found that the experimental group had an increase in moderate physical activity levels over baseline relative to controls (*p*-value = 0.03), with average increases of physical activity by 2 h and 18 min per week. The three arm intervention in the Prestwich *et al.* study [39] found that 42% of the intention and goal group receiving text messages had increased their physical activity time by 2 h per week relative to baseline. Similarly, 45% of the intention and plan text messaging group had increased their physical activity levels by 2 h per week over baseline levels. By contrast, only 22% of controls increased their physical activity when compared with baseline. Differences between group arms were statistically significant (*p*-values < 0.01). Increases in intentional physical activity levels were also found in the study by Turner-Mcgrievy *et al.* [35] where the experimental group receiving a podcast with an additional mobile device had physical activity levels of 196.4 kcal/day (SD = 45.9) relative to the control group receiving a podcast who engaged in physical activity levels of 100.9 kcal/day (SD = 45.1) (*p*-value = 0.02). The study by Shapiro *et al.* [33] measured physical activity by step counts and found that the experimental group receiving daily interactive SMS and MMS had increased their steps to 3000 steps per day (*p*-value < 0.05) relative to controls receiving monthly newsletters. They also found that there was a direct relationship between increased step counts and increased weight loss (*p*-value < 0.05). In a study comparing an enhanced podcast designed on social cognitive theory with a standard podcast without a theoretical basis, the experimental enhanced podcast group increased their reported physical activity levels by 0.8 days per week (SD = 0.9) relative to baseline, while the control group decreased their physical activity levels by 0.4 days per week (SD = 1.4) relative to baseline [35]. The differences between groups in reported physical activity levels were significant, favouring the experimental group (*p*-value < 0.01) [35].

### 3.9. Weight Measures

Weight loss was usually measured as changes in weight in kilograms or lbs. Some interventions provided pre and post changes in BMI measured in kg/m^2^ by measuring height via a stadiometer and weight by a weighing scale. The studies which reported change in body fat percentile were less common. The method of measuring weight across studies was valid, with weight being measured by objective digital weight scales. Body fat was measured using electrical impedance scales. Some studies examined changes in weight circumference measured in cm, utilizing a tape. Weight loss was measured by study staff, and frequency was often twice at baseline and post-intervention. However, some studies employed subject self-report of weight change, but this was not used alone without more objective measures by study staff. In addition to weight loss, some studies also measured cognitive process changes underlying weight loss and behaviours such as changes in self-efficacy to lose weight.

### 3.10. Weight Loss Overview

Positive changes in weight loss were observed across most studies in the intervention groups with mobile devices compared to baseline weight [30,31,32,33,35,37,38,39,40,41]. However, a few studies did not find significant between group differences in weight loss [33,34,36,42].

#### 3.10.1. Changes in Weight Mobile Phones

A total of 6 out of the 8 (75%) mobile phones interventions found significant changes in weight favouring the mobile phone intervention groups over the controls. Two studies did not have significant findings [33,36]. The study by Haapala *et al.* [30] found that subjects in the intervention group receiving a mobile text message intervention lost 4.5 kg over the 12 month study period from baseline weight (*p-*value < 0.01). The control group without an intervention also lost weight, but this was not as marked, with a mean weight loss of 1.1 kg. The differences in weight loss between the two groups were significant (*p*-value < 0.006). After adjusting for the variables age and sex, the Patrick *et al.* [32] study found that subjects in the intervention group receiving daily mobile phone SMS and MMS messages had lost 4.62 kg over the study period from weight at baseline. The control group receiving monthly health newsletters lost 0.17 kg over the study period compared to weight at baseline. After adjusting for the variables age and sex, the differences in weight loss between the experimental and control groups were 2.88 kg (*p*-value = 0.02), a 3.16% difference in weight loss between groups [32]. The three arm intervention by Carter *et al.* [38] found that the mobile phone group using an app to self-monitor weight lost the most weight from baseline of 4.6 kg (95% CI = −6.2–3.0). The diary group lost 2.9 kg (95% CI = −4.7–1.1) and the website group lost 1.3 kg (95% CI = −2.7–0.1). The Prestwich *et al.* study [39] found that subjects in the implementation intentions goal reminder group lost the most weight (0.53 kg) relative to the implementation intentions plan reminder group which gained 0.10 kg and the control group which lost 0.14 kg. The differences between the groups favouring the implementation intentions goal reminder group were significant (*p*-value = 0.046) (95% CI = 0.03–0.72). The study by Napolitano [40] *et al.* found that subjects in the text message and Facebook intervention lost 2.5 kg (SD = 0.4) from baseline. The Facebook only group lost 0.63 kg (SD = 2.4) from baseline. The differences between the two groups were marginally significant (*p*-value = 0.05).

#### 3.10.2. Changes in Weight Other Mobile Devices

A total of three out of four of the interventions employing mobile devices other than mobile phones had significant findings, favouring the intervention [35,37,41]. The study by Spring *et al.* [37] found that subjects utilizing PDA’s for self-monitoring of weight lost 6.3 lbs. (95% CI = −1.0–13.6) and the control group without a PDA lost 0.05 lbs. (95% CI = −4.7–4.6) at 12 months. The study by Burke *et al.* [41] found that subjects in the PDA with feedback intervention arm lost 2.32 kg over baseline (95% CI = −4.29–0.35) and this change was significant (*p*-value = 0.02). The PDA only group lost 1.38 kg (95% CI = −3.88–0.62) and the paper self-monitoring group lost −1.94 kg (95% CI 3.88–0.62), but these changes were not significant. The intervention did not find significant between group differences at 24 months [41]. The study by Turner-Mcgrievy *et al.* [34] found that subjects exposed to an enhanced podcast designed on social cognitive theory through either an intervention medium of an Mp3 player or iPod lost 2.9 kg from baseline weight (SD = 3.5). By contrast, the control group receiving a standard podcast without a theoretical foundation lost 0.3 kg from baseline (SD = 2.1). However, the addition of an extra second mobile device app for self-monitoring to the podcasting mobile component in the 2011 follow-up study [35] did not result in significant differences in weight loss between the groups (*p*-value > 0.98).

#### 3.10.3. Weight Loss and Adherence

Two studies examined the relationship between adherence to the weight loss intervention and subsequent weight loss [33,41]. Higher levels of adherence were associated with increased weight loss in study [33] but not in study [41].

### 3.11. Changes in BMI

The three studies which reported pre and post intervention changes in BMI all had results favouring the mobile device intervention groups. Study [38] found that the intervention group assigned to a Smartphone reduced their BMI by 1.6 kg/m^2^ (95% CI = −2.2–1.1). The web only group reduced their BMI by 0.5 kg/m^2^ (95% CI = −0.9–0.0) and the diary group by 1.0 kg/m^2^ (95% CI = −1.6–0.4). Study [34] found that subjects in the intervention groups reduced their BMI by 1.0 kg/m^2^ (SD = 1.2) and the controls by 0.1 kg/m^2^ (SD = 0.7), with significant between group differences (*p*-value < 0.001). Similarly, study [35] found that BMI reductions in the intervention group were greater than in the control group and that this difference was significant (*p*-value < 0.02).

### 3.12. Changes in Waist Circumference

The two studies measuring changes in waist circumference found positive reductions favouring the mobile device intervention groups [30,41]. The Haapala et alStudy [30] found intervention subjects reduced their waist circumference by 0.6 cm (SD = 1.7) and the control group by 0.4 cm (SD = 6.6). The Burke *et al* Study [41] found that the PDA with feedback group had reduced their waist circumference percentage by the most, 6.4% (95% CI = −11.5–1.8), and the PDA only and control groups reduced their waist circumference by 5.0% (95% CI = −8.5–1.7) and 4.0% (95% CI = −8.4–0.0), respectively.

### 3.13. Changes in Body Fat Percentage

The two studies measuring changes in percentage body fat both had positive statistically significant findings favouring the mobile device intervention groups [31,38]. The Hurling *et al.* study [31] found that the experimental group lost an average of 2.18% (SD = 0.59) body fat relative to the control group which lost 0.17% (SD = 0.81) body fat and that group differences were significant (*p*-value = 0.04). The Carter *et al.* study [38] found that the experimental group receiving the smartphone intervention lost (−) 1.3% body fat (95% CI = −1.70–0.8), while the diary control group lost 0.09% (95% CI = −1.5–0.4). The web group lost a total 0.5% body fat (95% = −0.90–0.0).

### 3.14. Study Quality

Study quality is summarized in Table 2. A total of 8 out of the 12 interventions had an adequate form of randomization [31,32,34,36,37,38,39,41]. The remaining four studies did not explain the form of randomization used. The forms of randomization used were often simple and stratified randomization. Block randomization and randomization by the process of minimization were also used, often employing a computer generated algorithm. Seven out of the 12 studies explicitly stated that allocation was concealed [32,33,34,35,36,38,39]. The remaining interventions did not provide information on allocation concealment. There were no significant baseline differences in characteristics of the intervention and control subjects across the studies. Study [32] is an exception, with differences in the age of participants. A total of 7 out of the 12 studies had a power and sample size calculation. Seven studies calculated sample size in accordance with a power of 80% to detect a notable difference between groups, often accounting for up to 30% attrition [30,33,34,36,37,39,41]. According to the Cochrane handbook [29], studies with retention over 80% are classified as having low attrition and studies with retention between 60%–79% are classified as having moderate attrition. Most studies had <30% attrition. The lowest reported attrition was 4% [40] and the highest overall was 38.8% [32]. Additionally, study [32] had unequal attrition between groups. All of the studies had analysed the groups by intention to treat analyses in accordance with original assignment, with some interventions conducting both ITT and completers analyses. Due to the nature of mobile devices, subject blinding was often not possible across the interventions. Subjects were blinded in one study by not knowing which podcast they were assigned to until the end of the intervention [35]. Assessors were blinded in three studies [30,38,39] and caregivers in one study [30]. Caregivers and assessors in the remaining studies were either not blinded or information was not explicitly provided on blinding status.

Intervention adherences across the studies were variable, with some studies not reporting adherence or direct measures of adherence in percentage of adherent participants. Adherence was measured in terms of compliance with self-monitoring or weight reporting [30,36,38,41]. Adherence was also measured according to frequency of group session attendance [37]. Study [37] did not find differences in group session attendance between intervention and control subjects. However, higher adherence was associated with increased weight loss [37]. Study [33] had an overall adherence of 69%, with no group differences in adherence. Study [38] found differences in adherence between groups. Additionally, study [36] found low levels of adherence to the intervention, with 54% of prompts receiving a response. There was a general trend of high adherence at the beginning of the interventions, followed by an interaction with the variable time, whereby adherence would decrease as a function of increased length of trial duration. This was observed across five studies [30,36,38,41]. In addition to this, the intervention groups were often reported to be more adherent than the controls groups [36,38,41]. Subjects in the smartphone intervention group were adherent for 92 days relative to 35 days for website controls in study [38]. Study [34] did not find differences in reported adherence to dietary and physical activity self-monitoring between groups, but did find differences in method of self-monitoring, with the intervention group being three fold more likely to utilize an app for self-monitoring [34].

### 3.15. Risk of Bias Grading

Risk of bias grading is summarized in Table 3. Based on the quality assessment table, risk of bias was graded according to the Cochrane recommended bias grading as low, high, or unknown [28]. A total of 8 out of 12 (67%) of studies had an adequate sequence generation [31,32,34,36,39,41] and 7 out of 12 (58%) of studies reported allocation concealment [32,33,34,35,38,39]. They were graded as having a low risk of selection bias. Four out of the 12 interventions were classified as having a low risk of detection bias by explicitly describing the blinding of outcome assessors [30,35,36,38], with the remaining being classified as having a high risk of bias by either not reporting this or leaving the answer unclear. Only one study was classified as having a low risk of performance bias as subjects were blinded. All studies were graded as having a low risk of attrition bias as they had acceptable levels of attrition (low to moderate) described earlier and were mostly intention to treat analyses. Due to the nature of mobile devices, blinding subjects may not always be possible and the handbook advises to assess the relative importance of a given domain in accordance with the intervention under investigation. Overall, half of the studies were graded as having a low risk of bias by meeting at the least 3 of the 5 domains.

**Table 2:** Critical Appraisal Trial Quality Rating [28,29].

Study	Randomization Method Clear + Appropriate?	Allocation Concealment?	No Significant Baseline Difference in Characteristics?	Assessors / Caregivers Blind to intervention?	Methods of data collection Valid?	Minimal attrition? Differences between groups?	Sample Size/Power Calculation	Subjects blind to intervention?	Intention to treat analysis?
Shapiro *et al.* 2012 [33]	N/A	√	√	- No	√	√	√	- No	√
					Objective weight scales and PA measures (CW series pedometer)	Attrition = 24%	N increased to 170 to allow for 25% attrition, 85% power		
Carter *et al.* 2013 [38]	√	√	√	√	√	√	-	- No	√
	Process of minimization			Fieldworkers undertaking measurement blinded	Objective portable weight scales	Attrition = 38.3% overall No- there were Significant group differences ( *p* -value = 0.01)	No Not a phase three trial		
Haapala *et al.* 2009 [30]	N/A	N/A	√	√	No- Self-report PA levels + diet But weight measured objectively in clinics (3×)	√	80% power N increased to 157 for 30% attrition	- No	√
				Nurse (caregiver) /weight outcome assessor blinded		27% -No Group differences in attrition			
Patrick *et al.* 2009 [32]	√	√	No Differences in mean age	No	√	√	-N/A	- No	√
	Simple Randomization	Allocation concealment at baseline measures but not after			Objective calibrated weight scales measures in study office	EG = 18% CG = 15.5%			
Hurling *et al.* 2007 [31]	√	N/A	√	-No	√	√	-N/A	-No	√
	Random stratification				Accelerometer, electrical impedance scales and weight scales	100% assigned to EG and CG complete study			
**Study**	**Randomization Method Appropriate + Clear?**	**Allocation Concealment?**	**No Significant Differences in Baseline Characteristics?**	**Assessors/** **Caregivers** **Blind to Intervention?**	**Methods of Data Collection Valid?**	**Minimal Attrition? Differences between groups?**	**Sample Size/ Power Calculation?**	**Subjects Blind to Intervention?**	**Analysis by Assignment?**
Turner-Mcgrievy *et al.* 2011 [34]	√	√	√	-No	√	√	√	-No	√
	Computerized random number generation				Objective weighing scales in study sites	89.6% completed study 11% attrition EG 7% CG	80% power, 86 total, accounting for attrition = N = 95–100		
Turner-Mcgrievy *et al.* 2009 [35]	N/A	√	√	N/A	√	EG = 15% attrition CG = 20%	N/A	√	√
					Objective Digital weight scale But self-reported PA levels				
Prestwich *et al.* 2010 [39]	√	√	√	√	√	√	√	No	Analysis by assignment excluding implementation intention recall analysis *
	Computer randomization generation				Objective weight measures with digital scale baseline+ follow-up - But PA subjectively reported	6% attrition	80% power allowing for 5%–10% attrition N = 149		
Brindall *et al.* 2013 [36]	Computer generated randomization	√	√	N/A	√	Attrition = 24%	Power 80% recruit N = 30 accounting for 30% attrition	No	√
					Weighed with objective digital scale in study office				
Napolitano *et al.* 2013 [40]	N/A	N/A	√	No	√	√	N/A	No	√
					Objective weighing scales study offices	100% completed 4 week assessment; attrition at 8 weeks = 4%			
Spring *et al.* 2013 [37]	√	N/A	√	No	√	√	√ N = 150 for 80% power	No	√
	Random permuted blocks stratified by age				Objective Calibrated weight scales	26% attrition			
Burke *et al.* 2011,2012 [41,42]	√	N/A	√	No	√	√	N = 210 for 80% power	No	√
	Computer implemented minimization algorithm stratified by age				Objective Digital weight scale - but self-report PA, 24 h 2 food recall	14% attrition			

Adapted from [28,29] quality assessment trial rating as Weak, Moderate, and Strong; √ = moderate-strong ratings No = weak rating; Attrition Weak rating = attrition >40% Moderate = retention 60%–79% and attrition <40% Strong = retention 80%–100% and attrition <20%; NA = Not Available.

**Table 3:** Risk of bias grading adapted from The Cochrane handbook Trial Appraisal Higgins *et al.* [28].

Author	Patrick [32]	Haapala [30]	Hurling [31]	Brindal [36]	Turner-Mcgrievy [1]	Turner- Mcgrievy [2]	Prestwich [39]	Spring [37]	Burke [42]	Shapiro [33]	Carter
Random Sequence Generation	√	?	√	√	√	?	√	√	√	?	√
Allocation Concealment	√	?	?	√	√	√	√	?	?	√	√
Participant Blinding	-	-	-	-	-	√	-	-	-	-	-
Blinding of outcome assessment	-	√	?	√	-	-	√	-	-	-	√
Incomplete Outcome Data	√	√	√	√	√	√	√	√	√	√	√

√ = low risk of Bias; Minus symbol - = high risk of bias; question mark symbol? = unknown/unclear risk of bias.

**Figure 2:**
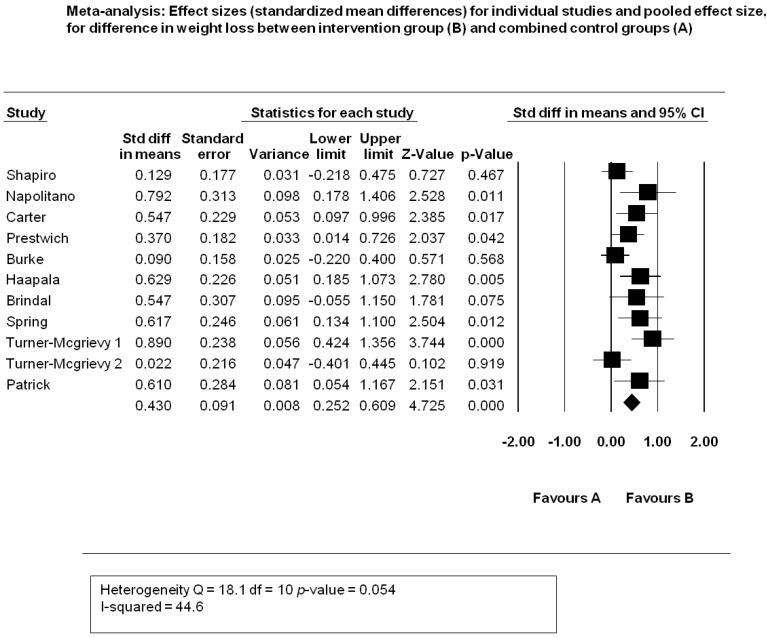
Mobile Devices and Weight loss Meta-analysis.

### 3.16. Meta-Analysis Weight Loss (kg)

#### 3.16.1. Overview

A total of 11 out of the 12 interventions were included in the meta-analysis. Study [41] was excluded as only post intervention changes in BMI were reported and the authors could not be contacted to determine weight change in kg.

#### 3.16.2. Results

According to the Cochrane handbook, medium effect sizes are values over 0.40 [29]. The results of the meta-analysis in Figure 2 indicate an overall medium effect size of 0.43 (95% CI = 0.252–0.609), favouring the intervention. The effect size was significant (*p*-value < 0.01).

#### 3.16.3. Heterogeneity

The results of the meta-analysis indicate moderate heterogeneity. I2 values of 30–60 indicate moderate heterogeneity, and >60 indicate high heterogeneity according to the Cochrane handbook. The Q statistic was 18.5 and the I2 45, indicating moderate heterogeneity. Moderate heterogeneity indicates that the results may slightly deviate or be inconsistent more from each other than they would by chance [29]. However, it was marginally non-significant (*p*-value = 0.054).

#### 3.16.4. Publication Bias

**Figure 3:**
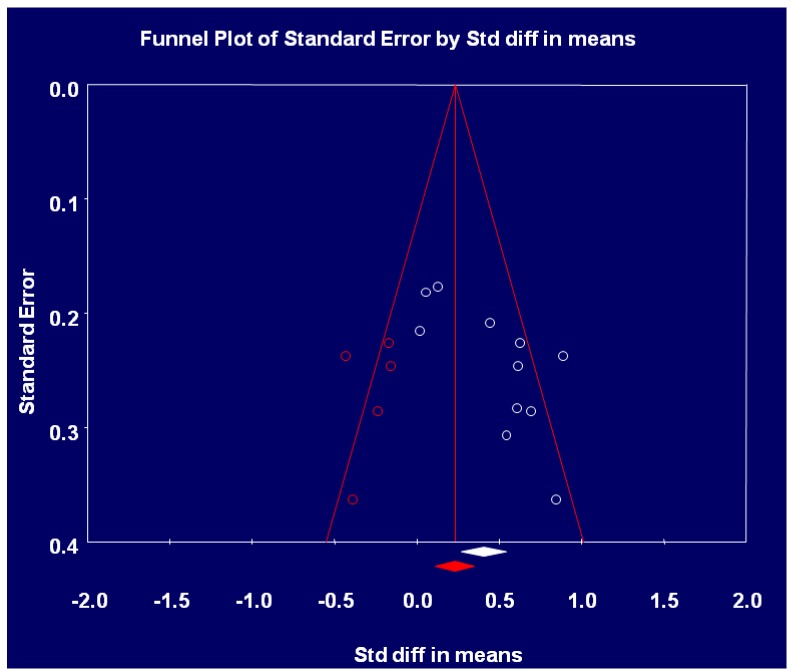
Funnel Plot for Publication Bias.

Assessment of publication bias is shown in the funnel plot in Figure 3. The funnel plot indicates some possibility of publication bias in both of the analyses due to the asymmetrical dispersion of effect points [29]. The funnel plot with imputation values in red demonstrates that small studies demarcated by large standard errors with positive effect sizes favouring the interventions were more likely to be published than studies with negative and less significant findings. However, the red imputation values for the overall effect size when taking into account symmetrical dispersion of effect points, indicates that the direction and size of the effect size would still be positive and significant (away from the null value of 0 for differences in means). Thus, in the absence of publication bias, the effect size would likely be smaller but still meaningful.

#### 3.16.5. Sensitivity Analysis

**Figure 4:**
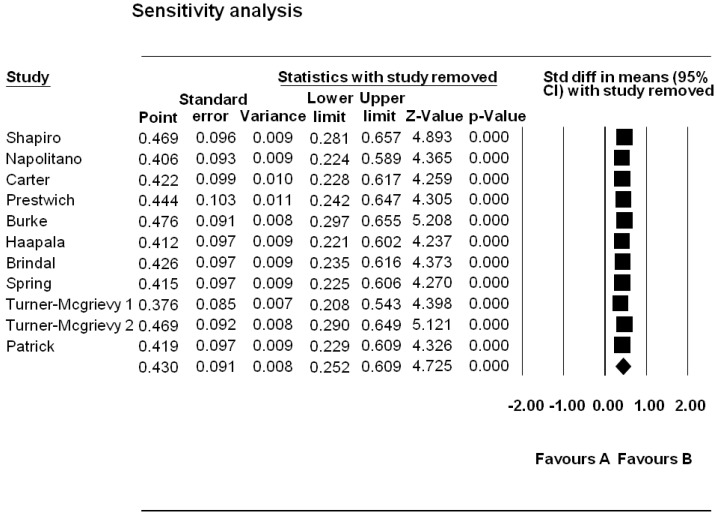
Sensitivity analysis.

The sensitivity analysis is demonstrated in Figure 4. The results of the sensitivity analysis indicate that the overall effect size does not very much when removing studies consecutively. The sensitivity analysis results indicate an overall similar effect size of 0.430 (95% CI 0.252–0.609) (*p*-value = 0.000) compared to the original effect size. This indicates that no intervention has a disproportionate effect on the results.

## 4. Results Part B: Intervention Content Analysis; Use of Theory and Behavior Change Techniques

### 4.1. Theoretical Base

Seven randomized controlled trials had an explicit theoretical base informing intervention design [31,33,34,35,36,39,41]. Some studies utilized more than one theory to inform the intervention [31,33,34,35]. The health psychology theories underpinning intervention design ranged from Implementation Intentions [39], Kanfer’s Self-regulation Model [41], aspects of Social Cognitive Theory [33,34,35], Elaboration Likelihood Model, Bagozzi’s Goals Theory, Decisional Balance [31], and Self-Monitoring (part of Control Theory) [32]. Social Cognitive Theory was one of the most common theories informing intervention design, with three studies explicitly employing it [33,34,35]. The interventions of three studies were also informed by the Elaboration Likelihood Model [31,34,35]. One study utilized Self-Efficacy Theory with Contingency Theory [30]. The Self-Monitoring Theory was explicitly used in one study [32]. Another study used the Health Action Model to inform the intervention [36].Whilst the remaining 3 studies did not have an explicit description of the theory used to inform intervention design, they nonetheless had utilized intervention techniques which were directly or partly linked with a theory. The studies [37,38,40] all involved aspects of self-monitoring, feedback, and goal setting which are techniques associated with Control Theory [11]. Lastly, the intervention by study [30] sought to prime self-efficacy which is associated with Social Cognitive Theory [11].

### 4.2. Predictors/Constructs

Ten interventions explicitly described a construct or predictor of behaviour change associated with a selected theory and intervention. Only one study provided a detailed diagram of how the intervention influences the target construct [34]. Four out of twelve studies measured a predictor of physical activity or dietary behaviour at baseline and post intervention follow-up. A fifth study measured predictors at week 4 and post-intervention at week 8 [36]. Positive findings in several mediators along the behaviour change to weight loss pathway were found in all seven studies [31,32,33,34,36,39,41]. The most common predictors described and measured were self-efficacy [33,34,40], user control [31,33], intentions [31,39], positive affect [30,36], and elaboration [34,35].

#### 4.2.1. Intentions and Sense of Control

The study by Hurling *et al.* [31] found that the experimental group receiving a mobile phone and web intervention had increased levels of perceived control to exercise over their baseline levels by 0.57 points and had increased intentions to exercise over baseline levels by 0.45 points relative to controls without an intervention [31]. Differences between the intervention and control groups were significant (*p*-value < 0.001) [31]. The intervention by Turner-Mcgrievy *et al.* [35] found a higher user control score the end of the 3 month intervention in the enhanced podcast group relative to the standard podcast controls by 0.13 points, and that differences between groups were significant (*p*-value = 0.001). However, a follow-up study comparing two enhanced podcasts, with the addition of a second mobile device to the intervention group, found that the user control score only increased during the first 3 months of the study, with group differences being non-significant at 6 months (*p*-value = 0.08).

#### 4.2.2. Positive Affect

Study [36] found that positive affect increased more in the intervention group by 0.10 points relative to controls with negative positive affect scores of −0.01 (SD = 0.13), and differences between groups were significant (*p*-value = 0.012).

#### 4.2.3. Self-Efficacy

One study did not find improvements in self-efficacy to lose weight and exercise [40]. Study [30] found that users had increased self-efficacy only if the 5% weight loss threshold had been reached, but this was not significant (*p*-value = 0.46), with the remaining subjects experiencing reductions in self-efficacy (*p*-value = 0.008) [30]. Study [34] found an interaction between time and user control, with initial increases in sense of control during the first 3 months, but this was not significant at 6 months (*p*-value = 0.08).

#### 4.2.4. Elaboration and Reduced Cognitive Load

The two podcasting studies [34,35] measuring changes in elaboration and cognitive load scores, both found favourable changes in elaboration and cognitive scores, and that differences between the intervention and control groups were significant (*p*-values < 0.05). Although elaboration scores were two fold higher at 3 months (41 SD = 12 *vs.* 24 SD = 15) in the intervention *versus* control groups, differences between groups were marginally non-significant at 6 months in the follow-up study (*p*-value = 0.06).

### 4.3. Intervention Components

#### 4.3.1. Text Message and App Component

The intervention components are summarized in Table 4. Seven out of the twelve mobile device trials had a text messaging component [30,31,32,33,38,39,40]. Text messages were frequently personally relevant according to individual progress and barriers [30,31,32,38,40]. They were also often tailored by enabling subjects to adjust message delivery in accordance with their personal schedule and time of the day [32,39]. Most text messages were interactive, requiring a user response [30,32,33], with one study requiring users to respond to 50% of the text messages [32]. User responses often involved progress content, but one study additionally employed knowledge based questions which required responses [33]. Text message frequency varied from 2–5 day [32,33], to once a month [31]. A mobile app component was used in four studies [30,34,36,38]. Text message functional purpose varied across studies and included the provision of health education, self-monitoring, transmitting information on diet and PA to study staff, reception of feedback on performance, and reception of motivational messages [30,31,32,33,38,39,40]. Mobile apps were used for self-monitoring of diet and physical activity [30,34,36,38] and feedback was provided via prompts on the smartphone dashboard [36]. One app was used for meal replacement purposes rather than caloric reduction monitoring [36].

#### 4.3.2. Health Education Component

Most of the interventions had a health education component. Health education included the provision of health newsletters, links to health education sites, and integration of health education into the intervention medium. The studies which provided details on content included the provision of tips on healthy eating [33], portion control [32], strategies for eating out [32], healthy food and physical activity environments [32], information on muscle strength training [32], recipe tips with meal suggestions [32], and information on government recommended levels of physical activity [39]. Mass media health education was used in the study by Turner-Mcgrievy [34], with soap opera podcasts serving as a medium for health knowledge and behaviour change. Frequency and variation of health education was only reported in one study which described five weekly alternating health education topics [32].

#### 4.3.3. Professional Support Component

Support from a health professional was utilized in three interventions. Mode of professional support delivery included in person support [37], over the telephone [32,37], and online support via Twitter [34]. Duration of professional contact ranged from 5–15 min [32] to in person professional sessions lasting 1.5 h [37]. Frequency of professional contact ranged from bi-monthly [37] to once a month [32]. Types of health professionals involved in the interventions included nutritionists, psychologists, dieticians [37], and counsellors [32].

**Table 4:** Intervention Components.

Study	Text Message Component?	App Component?	Health Education Diet/PA Component?	Self-Monitoring with Feedback?	Prompting/ Priming motivation, positive behavioural beliefs, or self- efficacy?	Professional Support Component?	Web-Component?	Comparator group without Intervention?	Use of single Intervention/ technology?
Carter *et al.* 2013 [38]	√	√	No	√	√	No	√	No Controls with diary or website interventionBut no mobile phone technology	√
	Personal Relevance Text message according to personal progress	My Meal Mate		App enables goal setting +self-monitoring via texts Feedback on energy +caloric expenditure	Text messages reinforce positive behavioural beliefs, confidence + motivation				Specific to mobile phones
Shapiro *et al.* 2012 [33]	√	No	√	Self-monitoring with pedometer, feedback with step graphical MMS charts	√	No	√	√	No, besides mobile phone intervention—access to e-newsletters
	Interactive with required text answers to knowledge based questions		Access to educational e-newsletters on Diet/PA Website health tips, nutrition recipes Knowledge-based texts		Text messages with personal motivating messages			Except health information	
Turner-Mcgrievy *et al.* 2011 [34]	No text message component	√	√	Goal setting activity podcast Self-monitoring app Feedback via Twitter	√	√	√	No, given podcast	No, podcast and mobile intervention for experimental group
		Fat Secret Calorie Counter App	Educational Podcasts on diet/PA		But no motivational mobile phone messaging podcast designed to enhance self-efficacy without positive reinforcing text messaging	Professional online support via Twitter			
**Study**	**Text message** **Component?**	**App Component?**	**Health Education Component?**	**Self-Monitoring with Feedback combined?**	**Positive Message Prompts or Motivational Component**	**Professional Support Component?**	**Web Component?**	**Comparator without Intervention?**	**Single technological component?**
Patrick *et al.* 2009 [32]	√	No	Education on Portion Control, Eating Out, meal planning, calorie education, strength training, exercise environments+Printed health education materials on diet + PA	√	√	√	No	No, Control group had printed monthly health education but not technology	No, experimental group had professional consultation phone calls and printed health education in additional to mobile phone intervention
	Individually tailored messages tailored to schedule and preference time+Interactive, with 50% of texts requiring a reply+Personally relevant messages according to dietary behaviour and change				Positive reinforcing text messages	Monthly phone calls from professional 5–15 min			
Haapala *et al.* 2009 [30]	√	√	√	√	No	No	√	√	√
	Personally relevant text messages according to % dieters reach daily’s target, personal calorie aims relative to current + Interactive text messages requiring replies		Offered web links to reliable diet + PA information	Goal settingFeedback via texts extent target met			Web dietary record keeping		Mobile phone exclusive intervention in experimental group
Hurling *et al.* 2009 [31]	√	No	No	√	√	No	√	√	No
	tailored texts based on perceived barriers			Scheduled weekly exercise goals Feedback via internet	3 Motivational benefits, motivating tips matched to each participant’s PA levels Email prompts				Mobile phone with internet as one intervention but social support online may be classified as adjunct intervention
Turner-Mcgrievy *et al.* 2009 [35]	No Text Messaging	No	√	No	√	No	No	No controls given standard podcast	√
			Health information diet+ PA via podcast + soap opera on podcast	Only end of podcast goal setting without feedback	Podcast designed to prime self efficacy + behavioural capability but No direct personal motivating /positive reinforcing messages				Specific to podcasts
Prestwich *et al.* 2010 [39]	√	No	√	No	No	No	No	√	√
	tailored by individual timing and delivery of message -Not personally relevant		Health information on government recommended PA levels, benefits +tips	Goal setting with self-monitoring but No feedback except goal and plan reminders	Only reminders to record goals/plan No personal motivating messages				Specific to SMS
**Study**	**Text Message Component?**	**App Component?**	**Health Education Component?**	**Self-Monitoring with Feedback?**	**Motivational Component?**	**Professional Support Component?**	**Web Component?**	**Control without intervention?**	**Single Technological Component?**
Brindall *et al.* 2013 [36]	No text messaging But app messages tailored to schedule	√	√	√	√	No	No	No given standard APP	√
		MRP app	Health information in app	App prompts self-monitoring with feedback on dashboard	Message board on iPhone provides motivational messages + trophy room				Specific to iPhone App
Napolitano *et al.* 2013 [40]	Tailored according to barriers	No app	√	√	Positive reinforcing text messages	No Buddy social support	√	√	No included Facebook intervention combined with text messaging
			5 health education information topics weekly	text message topics focus on goal setting with brief feedback			Facebook group		
Spring *et al.* 2013 [37]	No	No	√	√	No technology based motivational messages; over phone coach counselling	√	No	No controls had Move sessions	No, phone counselling and group sessions
						Yes in person dieticians, psychologists and phone counselling			
Burke *et al.* 2011, 2012 [41,42]	No	No	√	√	No motivational messages	No	No	No controls had either paper diary or PDA without feedback (group 2)	No group sessions with self-monitoring method

#### 4.3.4. Web Component

Half of the interventions had a web component [30,31,33,34,38,40]. Interventions which included internet supported participant login were not classified as web-based. Web-based interventions included ones which utilized the internet for self-monitoring, social support, and professional support. 

#### 4.3.5. Technological Components

Less than half of the studies (total 5) exclusively employed a single technological mobile device intervention in isolation from other technological mediums and without combination with different types of interventions such as professional support [30,35,36,38,39].

#### 4.3.6. Comparator

A total of five studies utilized a control group without the provision of an intervention for this group [30,31,32,39,40]. Study [33] provided the control with simple health information. The remaining seven studies provided the comparator group with some form of intervention [33,34,35,36,37,38,41,42].

### 4.4. Behaviour Change Techniques

A total of 22 out of the 26 Behaviour Change Techniques were adopted across the various interventions, meeting the coding criteria for the 26 BCT’s designed by Michie and Abraham *et al.* 2009 [15,16]. These included the use of self-monitoring, feedback, setting goals, revision of goals, provision of general health information, prompting intention formation, setting graded tasks, prompting barrier identification, provision of instruction how to perform the target behaviour, provision of encouragement, modelling/demonstrating behaviour, provision of rewards, teaching to use prompts, prompting practice, usage of follow-up prompts, social comparison, planning social support, prompting self-talk, relapse prevention, and stress management. The BCT’s not employed in the interventions were provision of information on the consequences of behaviour, general information about others’ approval, time management planning, participant identification as a role model and advocate, and the use of motivational interviewing. The most common BCT’s across the range of interventions were self-monitoring, goal setting, feedback, provision of general health information, encouragement, prompting practice and social support. The number of behavioural change techniques adopted per individual intervention was variable. However, all interventions had a minimum of 5 behavioural change techniques [30,41]. The maximum number of behaviour change techniques used in an individual intervention was between 10–12 [36,40] out of the possible maximum of 26 techniques.

#### 4.4.1. Goal Setting, Self-Monitoring and Feedback

All studies included goal setting, self-monitoring, and feedback. The exception is lack of feedback in studies [34,35]. Feedback was provided through different sources of media such as web groups, social networking sites, app feedback on the smartphone dashboard, and phone call feedback. Self-monitoring of diet and physical activity was also employed using various mediums including the use of mobile apps, PDA’s, the web, sending text messages, and using pedometers and accelerometers.

#### 4.4.2. Social Support

Planning social support involves the use of a human social supportive element; stimulating positive behavioural change [15,16]. The Behaviour change technique of social support was used in less than half of the studies. Social support mediums included online support through online forums [38] and social networking such as Twitter [34], buddy assignment [40], and group sessions [37,40,41]. However, study [41] did not provide enough information to determine if the social meetings had supportive elements to them.

#### 4.4.3. Prompt Practice

Additionally, all of the mobile phone studies used the behavioural change technique of prompting practice of diet and physical activity through either text message prompts or prompts on the iPhone push board [30,31,32,33,38,39,40].

#### 4.4.4. Stress Management and Relapse Prevention

Only one study employed the BCT stress management and relapse prevention [40].

#### 4.4.5. Graded Tasks

The use of graded tasks involves making tasks increasingly difficult [15,16]. This was adopted in four studies [33,37,40,41]. Graded tasks were set for levels of difficulty associated with physical activity levels and gradual reduction of caloric intake. 

#### 4.4.6. Modelling/Demonstrating behaviour

Modelling behaviour was used in two studies via podcasting with soap opera accounts of behaviour change and providing information on behaviour change [34,35].

#### 4.4.7. Social Comparison

The BCT social comparison refers to the use of a human social element which enables an individual engaging in behavioural change to modify his/her behaviour through the process of comparison and modelling of behaviour [15,16]. This may be employed through videos, buddy groups, and group class for instance [15,16]. This technique was adopted in a few studies employing mediums such as podcasting and soap opera demonstrations [34,35], group classes [37,41], and buddy support systems [40]. However, it is unclear whether the two studies [37,41] had opportunities for social comparison in group sessions. It may be inferred from study [37] that Move fitness sessions and practicing self-monitoring techniques in study [40] provided some opportunity for social comparison.

#### 4.4.8. Barrier Identification

The BCT prompting barrier identification involves identifying and planning for potential obstacles which may impede behavioural change progress [15,16]. This technique was used in four interventions [32,33,36,40].

#### 4.4.9. Provision of Encouragement

According Abraham and Michie (2008) [15,16], the BCT of providing encouragement includes motivating or praising an individual for their performance and may also include techniques to enhance self-efficacy in the form of verbal persuasion. The BCT of provision of encouragement was used in half of the interventions [31,32,33,36,38,40]. Encouragement was prompted using text messages, emails, and iPhone message boards [31,36,38,39,40].

#### 4.4.10. Contingent Awards

One study utilized the behaviour change technique provision of contingent rewards by adopting a trophy room on the iPhone app [36].

#### 4.4.11. Prompt Intention Formation

One intervention focused on the exclusive us of the BCT of prompting intention formation in subjects who actively planned their physical activity [39]. Three studies were partly informed by the BCT intention formation by having pre-set time bound PA or calorie goals for participants on a weekly or monthly basis [30,32,40].

#### 4.4.12. Follow-Up Prompts

Only two studies employed the BCT of follow-up prompts through monthly coaching or counsellor calls [32,37].

#### 4.4.13. Provide Instructions

Four studies provided instruction in the form of tips for engaging in the target behaviour and weight loss [32,34,36,41]. This was often employed by text messages, notably in study [32] where participants were given instructions and tips tailored to their barriers that would assist with engaging in the target behaviour. The PDA study [41] provided subjects with training and instructions on how to self-monitor. The podcasting [34] study provided strategies for weight loss at the end of the podcast.

#### 4.4.14. Prompt Practice

The BCT prompting practice refers to building habits through practicing the desired behaviour [15,16]. This technique was used across all mobile phone studies through text message prompts and prompts on the iPhone push board which stimulated practice of engaging in the target behaviour.

## 5. Discussion Part A: Implications of Mobile Device Interventions for Weight Loss

### 5.1. Changes in Weight

First, the results of this review demonstrate that mobile devices are potential media for weight loss among overweight and obese individuals. The systematic review has shown that mobile devices induce weight loss relative to baseline levels. Weight loss between intervention and control groups also favoured mobile device interventions. The overall pooled effect size for the meta-analysis indicated a medium significant effect size of 0.43 (95% CI = 0.252–0.609) (*p*-value ≤ 0.01), favouring the intervention. Another way of interpreting this result involves a conversion from Cohen’s d to percentage of overlap between groups [47]. Using the tabular conversion in [47], an effect size of 0.40 indicates that 66% of the control group would have a mean weight loss value below the average weight loss in the intervention group. The results are also similar to the results found in the meta-analysis on mobile devices for physical activity by Fanning *et al.* [24]. They found an overall moderate significant effect of 0.54 (95% CI exclude 0 and *p*-vale < 0.05). However, the results of this review do need to be interpreted with caution as the funnel plot indicates some possibility of publication bias. Nonetheless, the imputed effect size in the absence of publication bias indicates that the overall effect size, while smaller, would likely still be away from the null, favouring mobile interventions for weight loss.

Due to the fact that only four interventions utilized standard controls with no intervention [30,31,39,40] a separate comparing mobile device interventions with standard only controls was not possible. The remaining studies had control groups utilizing diverse non-mobile interventions, including only web-based interventions such as Facebook and web-based diaries, paper based self-monitoring methods for weight loss, and in person group session weight loss controls [31,37,38,39,40,41]. Therefore, it was not possible to conduct analyses comparing mobile devices with a specific control group receiving a specific non-mobile intervention such as web-based interventions. Thus, the pooled interventions had diverse comparator groups, ranging from standard controls, to varying non-mobile device controls. Inferences that may be drawn from the meta-analysis are that overall, the pooled significant medium effect size favours mobile device intervention groups when compared with varying controls including standard no treatment as well as non-mobile device controls. It should be noted, however, that three interventions allocated mobile devices for weight loss to both the intervention and control groups, but two had a standard control third arm. In these studies, results favoured theory informed mobile devices over non theory informed mobile devices and mobile devices with feedback over mobile devices without feedback and standard controls [36,39,41].

### 5.2. Changes in BMI, Body Fat Percentage, and Waist Circumference

Second, mobile devices have been found to directly influence several indicators of weight loss including reductions in body fat percentage, BMI, and waist circumference in addition to weight loss in kg found in the meta-analysis [30,38,41]. These indicators were reduced when compared with baseline levels and were also more reduced when compared with controls.

### 5.3. Changes in Diet and Physical Activity Levels

Third, mobile devices have also been found to induce weight loss indirectly by improving the behavioural determinants of weight loss including diet and physical activity levels [31,34,39,43,45,46]. Increases in moderate to vigorous physical activity levels both in duration and frequency were found across the studies. Fruit and vegetable intake levels increased in most studies measuring them, excluding one [43]. Reductions in fat intake were not found in all of the interventions measuring changes in fat intake. One study which measured sugar intake changes found reductions in sugar intake levels [42]. Improvements were also found in overall healthy eating patterns and energy dense food consumption [30,43].

### 5.4. Intervention Feature Complexity

The results of this review also highlight the complexity inherent in mobile device interventions for weight loss. Many of the studies utilized more than one intervention alongside a mobile device. Positive changes in weight favouring the intervention groups with meaningful differences between the intervention and control groups were observed in studies utilizing a mobile intervention medium alone as well as complex interventions that had used a mobile intervention alongside another intervention such as a traditional weight loss class or professional support. This indicates that mobile devices may be used as complementary adjuncts, enhancing the potential effects of traditional weight loss strategies as well as be used as primary singular interventions for weight loss. In addition to this, mobile phone interventions restrained to only the phone had varying levels of complexity with respect to utilization of the smartphone features in the intervention. Most of the mobile phone intervention studies utilized SMS combined with mobile app programs, making it difficult to determine if specific features of the mobile phone were more effective for weight loss. However, the Prestwich *et al.* study [39] had an SMS exclusive weight loss intervention and Brindall *et al.* [36] had an app only intervention, without mobile SMS. Both studies had positive findings, indicating that mobile phone weight loss interventions may focus on app design or mobile SMS alone or in combination as a complementary intervention.

### 5.5. Clinical Significance

Although most studies found weight loss favouring the intervention groups, weight loss of 5% which is defined as the clinically significant weight loss threshold [23], was not observed across all studies. However, most studies were short in duration. The longest study was the SMART trial undertaken for 24 months [41]. The trial found meaningful differences in weight loss at the 5% level favouring the PDA with feedback group at 6 months. However, this was not sustained at 24 months [41]. By contrast, the 12 month intervention in the Spring *et al.* study [37] found that the odds of 5% weight loss were 6 fold higher in the PDA intervention receiving monthly coaching calls than in the control group. They did not find any interaction with the variable time. It would be of research interest to further determine if the clinically significant weight loss threshold of 5% would be reached in other studies of longer duration and if the addition of monthly counselling phone calls enhances weight loss.

### 5.6. Implications of Negative Findings

It should be noted that whilst most studies found significant differences in weight loss from baseline weight relative to follow-up, a few studies did not find significant between group differences in weight loss in the intervention *versus* control groups. In most of these studies, the control groups had some form of a technological intervention such as a standard podcast standard mobile app, and standard PDA [34,36,42]. This indicates that technologies such as enhanced apps and podcasts in the intervention arms did not increase weight loss over and above the effect of standard devices in the control arms [34,36]. Interestingly, while study [42] did not find significant between group differences in weight loss at 24 months, reductions in waist circumference were found, favouring the PDA with feedback intervention arm. It would be of research interest to determine if mobile device type influences form of weight change, with certain devices being more useful for different aspects of weight change such as waist circumference reduction or overall weight loss.

### 5.7. Importance of Comparator

When interpreting and evaluating the results of behaviour change interventions, Michie *et al.* [48] ascertain that there is a need to be cognisant of the conditions of the control group. They posit that results favouring the intervention group may be two fold greater when the control group is not given any form of intervention. Many of the reviewed studies which had positive findings provided controls with some form of intervention, and Michie *et al.* argue that under such circumstances, the results may be underestimated [48]. Similarly, the implications of the negative findings in studies [34,36] should involve a consideration of context. Interestingly, the only study with negative results whose control group did not receive an intervention was study [33]. The researchers argue that the design of the study was similar to study [32], which had positive findings. They postulate that the main difference in the latter intervention was that it targeted physical activity rather than diet with physical activity [43]. It would be of research interest to determine whether interventions by mobile device are more effective if they target both diet and physical activity. However, it should be noted that study [39] focused only on physical activity and had positive findings.

## 6. Discussion Part B: The Implications of Theory and Behaviour Change Techniques

### 6.1. Theory

The use of theory in the interventions is summarized in Table 5. The wide use and success with weight loss associated with interventions founded on the theory, emphasizes its importance and potential role for weight loss through mobile device mediums. Most studies were explicitly informed by theory. Moreover, several theories were often integrated to inform interventions. 

The results of the review are in congruence with a systematic review on web-based interventions for behaviour change which found that wide use of theory was associated with improved outcomes [49]. Whilst the use of theory appears to be important for weight loss, the employment of several theories in a given intervention did not always improve weight loss outcomes across the studies [31,36]. 

**Table 5:** Theory Coding adapted from Michie and Prestwich Theory Coding and Michie and Abraham Illustrative Theory Techniques [27].

Study	Explicit Theory informing intervention?	Explicit Single Theory?	Theory predictors used to select recipients?	Target/Construct of Theory Mentioned?	Adequate Description of how construct predicts behaviour?	Health behaviour predictor measured baseline and follow-up	Change in construct predicting health behaviour in support of Theory?	Techniques adopted specific to target construct?
Prestwich [39]	Yes	Yes Implementation Intentions	No	Yes Intentions Goal and Plan Recall	Yes	Yes	Yes Increased Recall in SMS groups	Yes
Hurling [31]	Yes	No Multiple theories Social Comparison, Decisional Balance, Elaboration Likelihood Model, and Goal Theory	No	Yes Intentions, Expectation, and Perceived Control	No	Yes	Yes Increased perceived control and intentions	Yes
Turner-Mcgrievy 2011 [34]	Yes	No Multiple Theories Central theory Social cognitive theory (with elements of Contingency, Elaboration likelihood and Expectancy theory)	No	Yes User control, Cognitive load Elaboration, Expectancies, self-efficacy, expectation	Yes (detailed in 2009 study) [35]	Yes	Yes Increased user control toward elaboration at 6 months Increased self-control at 3 months, but not at 6 months	Yes
Turner-Mcgrievy 2009 [35]	Yes	Yes Social Cognitive theory	No	Yes User control Elaboration	Yes	Yes	Yes User control increased at 3 months and elaboration	Yes
Haapala [30]	Yes	No Dual theory Self-efficacy + Contingency theory	No	Yes Self-efficacyAttitudes towards medium	yes	Yes	Yes Increased self-efficacy in those achieving 5% weight loss + positive attitudes	Yes
Patrick *et al* [32]	Yes	Yes Self-Monitoring theory (implicit control theory	No	Yes Self-efficacy Cognisance of food choices	Yes	N/A (only measures of weight and PA + diet)	N/A	Yes
Shapiro *et al* [33]	Yes	Yes Social Cognitive Theory	No	N/A	N/A (Description of evidence based technique s but no description of construct link)	N/A (Only weight and PA measures)	N/A(no measures on self-efficacy change)	Yes
Burke *et al* [42]	Yes	Yes Self-regulation Model	No	Yes Self-Monitoring	Somewhat (description of self-monitoring)	N/A (only weight loss, adherence, diet +PA)	Yes Self-monitoring increase associated with increased weight loss	yes
Brindal *et al* [36]	Yes	Yes Health Action Model Theory	No	Mood (positive affect) Motivation	Yes	Yes	Yes improvement in positive affect, but not motivation	Yes
Spring *et al* [37]	Implicit Control Theory							
Carter *et al* [38]	No (implicit theory)							
Napolitano *et al* [40]	No (implicit social comparison?)							

N/A = not available.

### 6.2. Predictors

In addition to this, there were positive changes in cognitive predictors of weight loss along the causal pathways targeted by the intervention which was informed by a theoretical base. These included improvements in intentions, user control, user elaboration, and positive affect [30,31,34,35,36,39]. This suggests that mobile devices may induce weight loss by priming these predictors by applying theory to interventions, which leads to behavioural change in diet and exercise with subsequent weight loss.

### 6.3. Interaction with Predictors

However, one study found an interaction between the variable time and self-efficacy to lose weight [35]. This indicates that during short term interventions, self-efficacy to lose weight increases in the intervention groups, but in longer duration interventions, it decreases. Some interventions utilized alternating intervention components and topics by week to ensure continual subject stimulation and interest. Thus, possible explanations for the observed reduced sense of self-efficacy over time could include a saturation of intervention efficacy and loss of subject interest over time. Consideration of similar strategies over longer duration interventions is of research interest. 

### 6.4. Research on Physiological Pathways

Although study [35] found that self-efficacy only increased in the short-term in the enhanced podcast group informed by social cognitive theory relative to the standard podcast group, a recent follow-up study on podcasts *versus* a web intervention with similar content found increased levels of sense of control to lose weight and perception of intervention novelty in the podcasting group relative to web controls [50]. Interestingly, they found that the theory driven mobile intervention which increased levels of the cognitive predictor also had direct measurable physiological effects on users. Sensory neuronal stimulation was found in the enhanced podcasting group in the form of increased sweating measured through electrical skin conductance tests [50]. No other studies have objectively measured biological changes in user response to mobile mediums. Understanding the biological mechanisms through which mobile devices and use of theory enhance weight loss and cognitive pathways influencing their determinants is of research interest.

### 6.5. Applied Theories Informing Intervention Design

#### 6.5.1. Common Theories

The most common theory was Bandura’s Social Cognitive Theory [33,34,35]. The primary focus of this theory is priming self-efficacy to engage in the target behaviour [11]. Three of the four pathways through which social cognitive theory primes self-efficacy according to Webb [11], were found in this review including personal behaviour change attempts, simulation of behavioural change and experiences of another, and the use of verbal persuasion. Personal behaviour change attempts through practice and experience were used in all studies which focused on social cognitive theory. For instance, the podcasting intervention by Turner-Mcgrievy *et al.* [35] tapped on priming of self-efficacy through podcasts including soap opera podcasts which provided participants with first hand experiences of other subjects engaging in weight loss. The intervention also provided a source of verbal persuasion, with modelling and demonstration of behaviour via podcasts.

In addition to this, Petty’s Elaboration Likelihood Model was also a leading theory informing intervention design. Webb argues that the two pathways or routes which result in behavioural change are a central route, whose impact potential is subjected to an individual’s motivational disposition towards behaviour change, and a peripheral route, whose impact is subjected to a given individual’s reception to heuristic cues [11]. According to Webb, these include whether the intervening source is received favourably by the target audience [11]. Webb [11] postulates that the ability of interventions employing this theory to successfully stimulate behavioural change is determined by a subject’s a priori motivational stance. Adoption of this theory was found in study [35] as both the central conscious route in tandem with the peripheral unconscious route to behaviour change were targeted through podcasting.

#### 6.5.2. Less Frequent Theories

Implementation Intentions Theory was less frequently found in this review. It contains both an aspect of goal setting in tandem with active planning concerning how the health behaviour will be initiated, the timing of behavioural change, and where the health behaviour will take place [11]. According to Webb [11] implementation intentions are often underutilized in interventions targeting addictive behaviours.

Interestingly, Implementations Intentions Theory was adopted in study [39], finding that subjects who formed implementation intentions using the behavioural change technique of prompting intention formation with goal reminders lost the most weight. This indicates that Implementation Intentions Theory may hold potential for physical activity related to weight loss interventions through mobile devices. 

Another less frequently encountered theory in this review was Kanfer’s Self-Regulation Theory (model of self-control). This theory, like the one described above, has not been utilized frequently in addictive behaviour interventions according to Webb [11]. Its central tenet is that concentration on one task such as a given behaviour in need of change may lead to a process known as ‘ego-depletion’ whereby a given subject’s inhibitory regulatory mechanisms are in a state of inertia, unable to control other aspects of behaviour [11]. The hallmark of this theory is to focus on preventing this depletion from occurring [11]. It is unclear how study [41] by Burke et al sought to prevent this from occurring in the intervention groups.

#### 6.5.3. Implicit Theory

Several of the studies which did not explicitly discuss the use of theory, had adopted techniques associated with Goal as well as Control Theories by implementing goal setting, self-monitoring, and feedback [11]. The former theory is founded on the belief that specific measurable goals are more effective than general goals and that increasing goal difficulty is associated with improved outcomes and performance [11]. Setting specific measurable and time goals was integral to all of the interventions. In some studies, there were pre-set goals, while in others, subjects determined their goals as well as their levels of difficulty. The latter theory is founded on the premise that once a given goal is set, a self-regulatory mechanism is activated whereby a given subject compares their behaviour or goal with a reference value and concurrently seeks to adjust his/her behaviour in accordance with the goal [11]. The latter theory has been described by Webb [1] as not often being overtly presented in intervention studies [11] and the findings of this review highlight this [37,38,40].

### 6.6. Behaviour Change Techniques

#### 6.6.1. Key Adopted Behaviour Change Techniques

The behaviour change techniques in the interventions are summarized in Table 6. The findings of this review suggest that behavioural change techniques coded according to the Michie and Abraham criteria [15,16] were widely adopted across the reviewed studies. Findings from a recent systematic review on web-based interventions by Michie and Abraham [50] found that interventions were more effective if they adopted several behavioural change techniques relative to studies utilizing less techniques (*p*-value < 0.01). Although widespread use of BCTs appears to be positively associated with weight loss by mobile devices, this relationship does not appear to be linear across the studies. The study by Brindall *et al.* [36] adopted the most behavioural change techniques out of the studies (N = 12), and did not find significant differences in weight between the control and intervention groups.

The most commonly adopted and universally observed techniques were goal setting with self-monitoring and subsequent feedback, highlighting their potential importance for mobile device behavioural weight loss interventions. All of the studies also provided some form of basic health information to study participants. However, the depth of health education was variable suggesting the need for more research examining the level of health education required to achieve knowledge translation and subsequent behavioural change promoting weight loss. The techniques setting graded tasks and prompting barrier identification were also used in many studies. It appears that step wise behavioural weight loss change and individually tailored messages in accordance with barriers may be effective techniques as positive findings were found throughout the studies which adopted them. Prompting practice and provision of encouragement were also very common behavioural change techniques, most often employed through the use of mobile phone text messaging to stimulate behavioural change in dietary and physical activity behaviours. It is difficult to discern which techniques had the most significant effect on weight as several control groups also utilized different combinations of these techniques. Nonetheless, the results of this review are in agreement with a recent systematic review on BCTs for physical activity, which found that self-monitoring and prompting practice were widely used and found that these techniques may be implemented into the successful design of physical activity interventions [26]. In addition to this, supplementary information obtained from the authors of study [37] indicates that the BCT social support was integral to the intervention, less adherent subjects to the Move sessions where social support was provided lost less weight.

**Table 6:** Application of Abraham and Michie *et al.* (2007) 26 Item Coding Manual for Behaviour Change Techniques [15,16].

Behaviour Change Technique	Haapala *et al.* [30]	Prestwich *et al.* [39]	Patrick *et al.* [32]	Turner-Mcgrievy *et al.* 2009 [35]	Turner Mcgrievy *et al.* 2011 [34]	Napolitano *et al.* 2013 [40]	Brindal *et al.* [36]	Shapiro *et al.* [33]	Carter *et al.* [39]	Hurling *et al.* [31]	Burke *et al* [42]	Spring *et al.* [37]	Author
Provide General Information on Behaviour Health Link or Health Education	√	√	√	√		√	√	√		√			
Provide Information on Consequences		+											
Provide Information about other’s ‘Approval													
Prompt Intention Formation		√	√			√	√			√			
Prompt Barrier Identification			√			√	√	√					
Provide General Encouragement			√			√	√	√	√	√			
Provide Instruction or tips			√		√		√				√		
Graded tasks											√	√	√
Model/ Demonstrate the Behaviour				√	√								
Prompt Specific Goal Setting	√	√	√	√	√	√	√	√	√	√	√	√	√
Prompt Review of Behavioural Goals							√						
Techniques	Haapala	Prestwich	Patrick	Turner-Mcgrievy1	Turner-Mcgrievy-2	Napolitano	Brindal	Shapiro	Carter	Hurling	Burke	Spring	
Prompt Self-Monitoring of Behaviour	√	√	√	√	√	√	√	√	√	√	√	√	
Provide Feedback on Performance	√	√	√			√	√	√	√	√	√		
Provide Contingent Rewards							√						
Teach to use Prompt Cues													
Agree Behavioural Contract		√											
Prompt Practice	√	√	√			√	√	√	√	√			
Use of Follow-up Prompts			√										
Provide Opportunity for Social Comparison				√	√	√							
Plan Social Support/Social Change					√	√			√	√			
Prompt Identification as a Role Model/Position Advocate													
Prompt Self Talk							√						
Relapse Prevention						√							
Stress Management						√							
Time Management													

Whilst all studies provided general health information, none of the studies provided information on the consequences of behaviour and it may be of research interest to determine if this additional technique may be useful. Furthermore, none of the studies adopted techniques to manage time and this may be a useful technique to consider for future interventions. Research suggests that often time management is a key barrier towards eating healthy and engaging in physical activity [51]. In addition to this, stress management and relapse prevention were only employed in a single study. It may be of research interest to determine whether these techniques may be useful for behavioural weight loss interventions.

#### 6.6.2. Diverse Media of BCT Delivery

The results of this review also suggest that the media through which BCTs were delivered varied significantly, suggesting that diverse media may be utilized to successfully deliver BCTs. These included various mobile device media as well as diverse electronic input and output functions associated with these media. They also included combinations of human contact with electronic media such as human BCT delivery through indirect as well as direct face-to-face sessions. For example, the use of social support through diverse electronic media such as Facebook, online groups, and in person group sessions suggests that this technique may be delivered by multiple mobile electronic modalities. Another example would be the use of modelling behaviour both through opera podcasts using mass media health promotion and through opportunities for behaviour modelling in group sessions.

### 6.7. Connection of Behavioural Change Techniques with Theory

Although all of the techniques underpin the health psychology theories described earlier, some studies utilized combinations of BCTs associated with a mix of various theories according to criteria in Michie and Abraham [52]. Often techniques were adopted that were associated with theory which implicitly informed the intervention such as elements of control theory [11].

This review also found that while intervention techniques illustrative of a theory were adopted, not all possible techniques associated with a given theory were utilized according to the Michie and Abraham list of possible techniques per theory [52].

**Figure 5:**
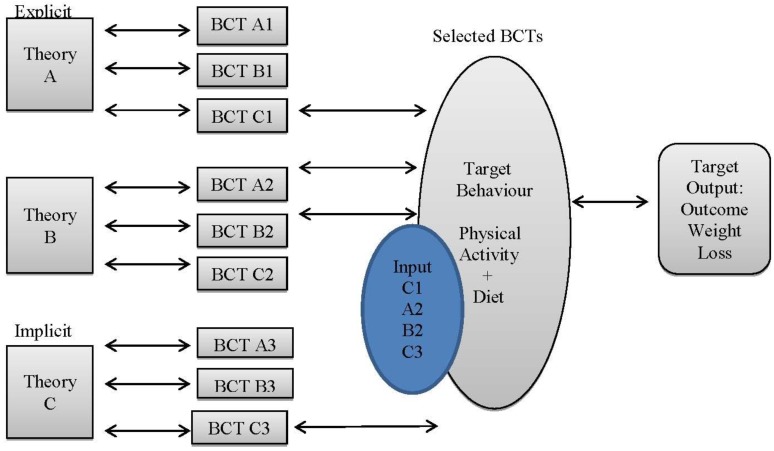
BCT and Theory Connection in Reviewed Trials.

Figure 5 adapted from information on theory coding and BCT linkage in Michie and Abraham [52] and applied to these findings, summarizes patterns of theory and BCT connections in this review. A given theory may have several behavioural change techniques as represented by the alpha numerical characters representing techniques A, B, and C. Not all interventions have adopted all techniques associated with a given theory as found in this review. Several theories can be applied to an intervention as found in this review, represented by A, B, and C. The use of theory found in this review may also be implicit or explicit. Several theories with select techniques may be adopted by an intervention, represented by the input function. The techniques then target the given behaviours such as physical activity and diet, with the output function of weight loss. For instance, social cognitive theory has elements of provision of instruction, general encouragement, barrier identification, and modelling of behaviour [52]. For example, an intervention may utilize only prompting encouragement from social cognitive theory, without other BCTs such as modelling of behaviour, and combine it with goal setting, self-monitoring, and feedback from control theory, which may or may not be explicitly mentioned.

## 7. Summary of Discussions Part A and B

### 7.1. Synopsis

As a review of the two discussion sections, a synopsis of this work will be recapitulated. The findings of this thesis are that mobile devices induce both direct and indirect positive changes in weight. They induce direct weight loss by reducing weight measured in kg, body mass index, waist circumference, and body fat percentage compared to baseline levels. They also induce more weight loss when compared with control groups. The pooled effect size in the meta-analysis indicates an overall medium significant effect of mobile devices on mean weight loss.

Throughout this systematic review, weight loss favouring mobile devices was found in most studies comparing subjects utilizing mobile devices for weight loss with standard controls. Weight loss favouring mobile devices interventions was also found in most studies comparing mobile device intervention groups with controls receiving diverse non-mobile weight loss interventions including in person face to face sessions, web-based interventions, and paper based self-monitoring interventions.

Mobile devices also influence weight indirectly by improving the behavioural determinants of obesity and overweight. Improvements in indirect indicators of weight loss were represented by increased levels of moderate to vigorous physical activity and improvements in dietary intake when compared with baseline scores and when compared with control groups.

Theory appears to play an important role in mobile device interventions as most interventions were either directly or indirectly informed by implicit elements of theory. Behaviour change techniques appear to play an important role in mobile device interventions as all interventions had a minimum of five techniques, but not all techniques illustrative of a theory were utilized. The most common techniques found were prompting encouragement, providing social support, prompting practice, and providing health information. Improvements in hypothesized predictors along the causal pathway were found for positive affect, elaboration, intentions, and self-control to lose weight post-intervention. This indicates that interventions informed by a theoretical base successfully targeted the theoretical construct hypothesized to stimulate changes in the behavioural determinants of obesity, leading to weight loss.

### 7.2. Strengths and Limitations

#### 7.2.1. Strengths

This review has a number of strengths. It provides updated data on weight loss to the early mobile device systematic review [24] by incorporating findings from the most modern devices such as smartphones and iPods. It further provides information on changes in the behavioural determinants of weight loss including diet and physical activity. Second, this is the first meta-analysis on mobile devices and weight loss. Third, this is the first review to utilize BCT coding and theory analysis for weight loss by mobile device.

#### 7.2.2. Limitations

There are several limitations to this review. Although the meta-analysis found a moderate effect size that was statistically significant, the results should be interpreted with caution due to the small number of studies and potential of publication bias. Furthermore, one intervention could not be added to the analysis. However, it is unlikely that it would have changed the direction of the effect size as it had positive findings. Additionally, many of the studies were of short duration, making it unclear if weight loss is sustained in the long-term. Whilst the clinically significant threshold for weight loss was not always met across the studies, there is a possibility that studies of longer duration may find clinically significant weight loss. More research is needed to determine this.

Many of the studies were of moderate quality. Concerns throughout this review were raised of potential biases notably detection and performance biases. With respect to the latter, the nature of mobile devices makes subject blinding difficult. Additionally, a few studies did not report whether allocation was concealed, giving rise to concerns of possible selection bias. Furthermore, not all studies reported intervention adherence. A few of the studies were pilot studies, without sample size and power calculations to detect meaningful differences when accounting for attrition. The limitations found in this review are common to the limitations found in similar systematic reviews on mobile device types and weight loss [23,24,25].

Another potential limitation of this review is that data were extracted by one reviewer and optimally, there should be more than a single reviewer. It is unlikely that BCT coding would have been affected by one coder as research suggests that the level of agreement between reviewers is high, with an average agreement of 93% [15]. The only exception pertains to the BCT prompting encouragement, which was found to have a kappa of 0.66, indicating 66% agreement between coders [15]. 

### 7.3. Future Directions

There is a need for interventions of longer duration to determine if weight loss is sustained in the long-term and to determine if more interventions meet the clinically significant 5% weight loss threshold. There is also a need for more interventions with a low risk of bias by meeting several key domains in the Cochrane handbook, notably outcome assessor blinding, clearly described methods of randomization, and reported allocation concealment. It is anticipated that with several protocols underway and emerging research in this area, future interventions will improve in these areas, increasing the robustness of the evidence base.

Several research questions have also been raised throughout this review described earlier and may be considered as follow-up research topics.

## 8. Conclusions

In summary, this review had two objectives, with the main primary central aim to determine whether mobile weight loss interventions induce weight loss and stimulate positive changes in weight related behaviours including diet and physical activity. The primary research question was:

*Do mobile devices induce weight loss and favourable changes in diet and physical activity when compared to baseline weight and scores? Do they induce weight loss when compared with standard controls receiving no intervention and or when compared with controls receiving non-mobile weight loss interventions*?

### 8.1. Primary Central Research Objective

In order to answer this question, a systematic review and meta-analysis were undertaken. First, this review found that weight loss interventions by a mobile device medium induce weight loss. The meta-analysis results favoured mobile device interventions for weight loss. The meta-analysis found an overall medium effect size of 0.430 (95% CI = 0.252–0.609) (*p*-value ≤ 0.01). Using the tabular conversion to percentage of overlap in [47], an effect size of 0.40 indicates that 66% of the control group would have a mean weight loss value below the average weight loss in the intervention group. Inferences that may be drawn from the meta-analysis are that overall, the pooled significant medium effect size favours mobile device intervention groups. Throughout this review, weight loss favouring mobile devices was found when comparing baseline weight with post-intervention weight as well as when comparing the amount of weight loss between intervention and control groups. The intervention groups were compared with varying controls including standard no treatment controls as well as controls receiving diverse non-mobile device weight loss interventions, indicating that mobile devices may be induce more favourable weight loss when compared with these groups. However, the interpretation of the overall weight loss has been warranted to be interpreted with caution due to the possibility of some publication bias. Nonetheless, the imputed effect size in the absence of publication bias indicates that the overall effect size, while smaller, would likely still be away from the null, favouring mobile interventions for weight loss.

Second, this review also found that diverse mobile devices induce positive reductions in waist circumference, BMI, and body fat percentage relative to baseline measures. Reductions in these indicators were also found when comparing mobile device intervention groups with both standard controls not receiving any treatment as well as diverse controls receiving different non-mobile weight loss interventions.

Third, this review found that mobile devices induce positive changes in weight related behaviours. These included increases in moderate to vigorous physical activity and improvements in dietary intake. Dietary intake improved when compared with baseline intake scores and when compared with controls. Physical activity levels also improved when compared with baseline physical activity levels and when compared with controls. Improvements were found for dietary intake of fruit and vegetables with the exception of study [43]. Improvements were also found in sugar intake levels and energy dense food consumptions scores.

The secondary aim of this thesis was to gain a greater understanding of the key theories and behavioural change techniques involved in informing mobile weight loss interventions. The secondary research question was:

What health psychology theories and psychological behaviour change techniques inform mobile device weight loss intervention design and are theoretical constructs along the causal pathway leading to weight loss improved post-intervention?

### 8.2. Secondary Research Objective

In order to answer this question, a systematic review with a theoretical analysis of coded data was undertaken.

First, this review found that the use of health psychology theory is widely adopted across the studies and may have an important role in the success of weight loss interventions. Most interventions were informed directly by an explicit theory or indirectly by implicit elements of a theory. The most common theories adopted across the studies included Social Cognitive Theory, Implementation Intentions Theory, Elaboration Likelihood Theory, Goal Theory, and Control Theory.

Second, this review also found that several behaviour change techniques derived from one theory or mixes of several theories were used. However, not all techniques illustrative of a theory were used. The behavioural change techniques adopted across the studies in descending order from the most common included goal setting, self-monitoring, feedback, prompting practice, providing general encouragement, providing social support, prompting barrier identification, providing instruction, providing opportunities for social comparison, relapse prevention, and stress management. The wide use of BCT’s indicates that they may play an important role in mobile device weight loss interventions. 

Third, improvements in constructs targeted by the interventions informed by a theoretical base were found. These included improvements in user control, positive affect, elaboration, and intentions when comparing baseline to post-intervention measures, suggesting that the interventions successfully targeted the predictor of primary interest.

Both the primary and secondary research questions were answered throughout this work. The combined results of both research questions have led to two central arguments and subcategory arguments of this work.

(1) This work has argued throughout that mobile devices induce positive changes in weight loss both directly and indirectly. They induce weight loss directly through reductions in weight in kg, body mass index, body fat percentage, and waist circumference. This was found in interventions comparing mobile devices with standard controls and controls receiving diverse non-mobile weight loss interventions. They also induce weight loss indirectly by improving the behavioural determinants associated with obesity including diet and physical activity; (2) Theory and behavioural change techniques appear to play an important role in mobile weight loss interventions, but not all possible techniques illustrative of a theory were utilized throughout the interventions. Theory informed interventions successfully led to improvements in most cognitive predictors along the causal pathway which are hypothesized to lead to improvements in the behavioural determinants associated with weight loss.

Drawing on a larger public health perspective, tackling the obesity and overweight pandemic requires efforts on the part of multiple sectors of society including the social, economic, political, and environmental dimensions [53]. The Dahlgreen and Whitehead (1991) [54] health promotion sphere may be applied to obesity and overweight. Whilst internal layers of the sphere such as age, sex, and genetics are non-modifiable, tackling external layers of the sphere in the form of lifestyle, policy including food taxation [55], equity in income distribution [54], green environmental space [56], and a sustainable agricultural environment [54] are all integral to reducing overweight and obesity. Weight loss by mobile devices rests within the lifestyle sphere of this model which may further be categorized into reflective process, requiring individual conscious choice and in tandem with automatic processes, which are non-conscious and require altering choice architecture [57]. Mobile weight loss interventions tap on reflective processes by stimulating behaviour change through informed choices and self-monitoring. They hold some potential as mediums for behaviour change both through their widespread population use and complexity of features that allow for the integration of numerous theoretical constructs and behaviour change techniques, particularly self-monitoring with timely feedback as demonstrated in this review. Under the conditional that emerging improved interventions with longer duration and improved methodology will demonstrate meaningful and sustained weight loss, then these interventions may be considered as part of the public health efforts in the health promotion sphere. They may hold promise as singular interventions for weight loss as well as integrated interventions as part of the broader varying efforts and strategies on the continuum described above which target the obesity and overweight conundrum.

## Appendix

**Table A1:** Summary of CINAHL Search via EbscoHost.

**PICO Definition** **Population–Humans > 18 years of age overweight or obese** **Intervention–Mobile Devices** **Control–standard treatment or no intervention** **Outcome–Weight Loss (Kg) or (lbs.)** **Study Design–Randomized Controlled Trial** **Search Options:** **Limiters-Linked Full Text; References Available; Scholarly (Peer Reviewed) Journals; Population Group: Human; Publication Type: Peer Reviewed Journal; English; Language: English; Population Group: Human; Document Type: Journal Article; Publication Type: Academic Journal; Document Type: Article; Language: English** **Narrow by Subject Age 0: adulthood (18 years and older)** **Search modes:** **Boolean phrase** **Device:** **Mobile Phone OR smartphone OR cellular phone AND Mobile device (N = 1851) without limiters** **With age limit 13–17 and 18+ and full text with reference limiters above in search options (N = 141)** **Text message* OR short message service OR SMS (N = 1131)** **With limiters N = (107)** **PDA OR Personal Digital Assistant OR palmtop (N = 947)With limiters specified above (N = 81)** **Outcome/Targets:** 4. **Weight loss OR weight control or weight reduction (N = 8474)** **With limiters (N = 814)** 5. **Obesity OR overweight (N = 17,554)** **With limiters (N = 1256)** 6. **1 and 5 (N = 6300)** **With limiters (N = 703)** 7. **1 and 4 and 5 (N = 9657)** **With limiters (N = 753)** **All terms with limiters** 8. **2 and 4 (N = 641)** 9. **2 and 5 (N = 584)** 10. **2 and 4 and 4 (N = 724)** 11. **1 and 2 and 5 (N = 1145)** 12. **1 and 2 and 4 and 5 (N = 731)** 13. **3 and 4 (N = 635)** 14. **3 and 4 and 5 (N = 1002)** 15. **1 and 2 and 3 and 4 (N = 683)** **Search Terminology:** **Full Large Search String (1 and 2 and 3 and 4 and 5)** **mobile phone OR smartphone OR cellular phone AND text message* OR short message service OR SMS OR mobile device AND PDA OR personal digital assistant OR palmtop AND Weight loss OR weight control OR weight reduction AND obesity OR overweight** **Search Options:** **Limiters–Linked Full Text; References Available; Scholarly (Peer Reviewed) Journals; Population Group: Human; Publication Type: Peer Reviewed Journal; English; Language: English; Population Group: Human; Document Type: Journal Article; Publication Type: Academic Journal; Document Type: Article; Language: English** **Narrow by Subject Age 0: adulthood (18 years and older)** **Search modes :** **Boolean/Phrase Results (N = 1162)** **Databases searched:** **PsychInfo (N = 1126)** **PyschArticles (N = 456)** **Library Information Science and Technology Abstracts (N = 57)**

## References

[1] The World Health Organization Global Health Observatory. Obesity situation and trends. http://www.who.int/gho/ncd/risk_factors/obesity_text/en/.

[2] Kelly T., Yang W., Chen C.S., Reynolds K., He J. (2008). Global burden of obesity in 2005 and projections to 2030. Int. J. Obes..

[3] (2012). The Global Burden of Disease Study 2010. Lancet.

[4] The World Health Organization (2013). Promoting fruit and vegetable consumption around the world. http://www.who.int/dietphysicalactivity/diet/en/index.html.

[5] The World Health Organization (2013). Physical activity. http://www.who.int/dietphysicalactivity/pa/en/index.html.

[6] Withrow D., Alter D.A. (2011). The economic burden of obesity worldwide: A systematic review of the direct costs of obesity. Obes. Rev..

[7] Vadon R. (2007). Cost of obesity overestimated. BBC news..

[8] The World Health Organization (2004). Global strategy on diet, physical activity, and health. http://www.who.int/dietphysicalactivity/en/.

[9] The World Health Organization (2004). Global recommendations on physical activity for health. http://www.who.int/dietphysicalactivity/publications/9789241599979/en/index.html.

[10] Food Standards Agency FSA nutrient and food based guidelines for UK institutions. http://www.food.gov.uk/.

[11] Webb T.L., Sniehotta F.F., Michie S. (2010). Using theories of behaviour change to inform Interventions for addictive behaviours. Addiction.

[12] Volkow N.D., Wise R.A. (2005). How can drug addiction help us understand obesity?. Nat. Neurosci..

[13] Tones K., Green J. (2004). Mass Communication and Community Action. Health Promotion: Planning and Strategies.

[14] Zimmerman G.L., Olsen C.G., Bosworth M.S. (2000). Stages of change approach to helping patients change behaviour. Am. Fam. Physician.

[15] Michie S., Richardson M., Johnston M., Abraham C., Francis J., Hardeman W. (2013). The behaviour change technique taxonomy (v1) of 93 hierarchically clustered techniques: Building on international consensus for the reporting of behaviour change interventions. Ann. Intern. Med..

[16] Abraham C., Michie S. (2007). A taxonomy of behaviour change techniques used in interventions. Health Psychol..

[17] Burke L., Wang J., Sevick M.A. (2011). Self-monitoring in weight loss: A systematic review of the literature. J. Am. Diet Assoc..

[18] Michie S., Abraham C., Whittington C., Mcateer J. (2009). Effective techniques in healthy eating and physical activity interventions: A meta-regression. Health Psychol..

[19] Coons M., Roehrig M., Spring B. (2002). The potential of virtual reality technologies to improve adherence to weight loss behaviours. J. Diabetes Sci. Technol..

[20] Lefebre C. (2009). Integrating cell phone and mobile technologies into public health practice a social marketing perspective. Health Promot. Pract..

[21] ITU World Telecommunications (2010). The world in 2010. The rise of 3G. http://www.itu.int/ITU-D/ict/material/FactsFigures2010.pdf.

[22] Whittaker R., Mcrobie H., Bullen C., Borland R., Rodgers A., Gu Y. (2012). Mobile-based interventions for smoking cessation. Cochrane Database Syst. Rev..

[23] Shaw R., Bosworth H. (2012). Short message service (SMS) text messaging as an intervention medium for weight loss: A literature review. Health Inform. J..

[24] Fanning J., Mullen S.P., McAuley F. (2012). Increasing physical activity with mobile devices: A meta-analysis. J. Med. Internet Res..

[25] Bacigalupo R., Cudd P., Littlewood P.C., Bissell M.S., Hawley S., Buckley Woods H. (2012). Interventions employing mobile technology for overweight and obesity: An early systematic review of randomized controlled trials. Obes. Rev..

[26] Bird E.L., Olgivie D., Powell J., Baker G., Mutrie N., Sahlqvist S. (2013). Behaviour change techniques used to promote walking and cycling: A systematic review. Health Psychol..

[27] Michie S., Prestwich A. (2010). Are interventions theory-based? Development of a theory coding scheme. Health Psychol..

[28] Higgins R., Altman D.G., Gotzsche P.C., Juni P., Moher D. (2011). The Cochrane Collaboration’s tool for assessing risk of bias in randomized trials. Br. Med. J..

[29] Higgins J.P.T., Green S. (2011). Cochrane Handbook for Systematic Reviews of Interventions. http://www.cochrane-handbook.org.

[30] Haapala I., Barengo N., Biggs S., Surakka L., Manninen P. (2009). Weight loss by mobile phone: A 1-year effectiveness study. Public Health Nutr..

[31] Hurling R., Catt M., de Boni M., Fairley B.W., Hurst T., Murray P., Richardson A., Sodhi J.S. (2007). Using internet and mobile phone technology to deliver an automated physical activity program: Randomized controlled trial. J. Med. Res..

[32] Patrick K., Raab F., Adams M.A., Dillon A., Zabinski M., Rock C.L., Griswold W.G., Norman G. (2009). A text-based intervention for weight loss. A randomized controlled trial. J. Med. Internet Res..

[33] Shapiro J.R., Koro T., Doran N., Thompson S., Sallis F.J., Calfas K., Patrick K. (2012). Text4diet: A randomized controlled study using text messaging for weight loss behaviours. Preventive Med..

[34] Turner-McGrievy G., Tate D. (2011). Tweets apps and pods: Results of the 6-month mobile pounds off digitally (mobile pod) randomized weight-loss intervention among adults. J. Med. Internet Res..

[35] Turner-McGrievy G., Campbell M.K., Crosby L. (2009). Pounds off Digitally: A randomized Podcasting weight loss intervention. Am. J. Preventive Med..

[36] Brindall E., Hendrie G., Freyne J., Coombe M., Berkovsky S., Noakes M. (2013). Design and pilot results of a mobile phone weight-loss application for women starting a meal replacement programme. J. Telemed. Telecare.

[37] Spring B., Duncan J., Janke A., Kozak A.T., Mcfadden G., DeMott A., Pictor A., Epstein L., Siddique J., Pellegrini C. (2013). Integrating technology into standard weight loss treatment: A randomized controlled trial. JAMA Internal Med..

[38] Carter M.C., Burley V.J., Nykjaer C., Cade J.E. (2013). Adherence to a smartphone application for weight loss compared to website and paper diary: Pilot randomized controlled trial. Med. Internet Res..

[39] Prestwich A., Perugini M., Hurling R. (2010). Can implementation intentions and text messages promote brisk walking? A randomized trial. Health Psychol..

[40] Napolitano M.A., Hayes S., Bennette G.G., Ives A.K., Foster G.D. (2013). Using Facebook and text messaging to deliver a weight loss program to college students. Obesity.

[41] Burke L., Styn M.A., Sereika S.M., Conroy M.B., Ye L., Glanz K., Sevick M.A., Ewing L.J. (2012). Using mHealth technology to enhance self-monitoring for weight loss a randomized Controlled trial. Am. J. Preventive Med..

[42] Burke L.E., Conroy M.B., Sereika S., Elci O.U., Styn M.A., Archaya S.D., Sevick M.A., Ewing L.J., Glanz K. (2011). The effects of electronic self-monitoring on weight loss and dietary intake: A randomized behavioural weight loss trial. Obesity.

[43] Norman G.J., Kolodziejczyk J.K., Adams M.A., Patrick K., Marshall S.J. (2013). Fruit and vegetable intake and eating behaviours mediate the effect of a randomized text-message based weight loss program. Preventive Med..

[44] Turner-McGrievy G., Beets M.W., Moore J.B., Kaczynski A.T., Barr-Anderson D.J., Tate D.F. (2013). Comparison of traditional *versus* mobile app self-monitoring of dietary intake and physical activity among overweight and obese adults participating in the mhealth weight loss program. Am. J. Med. Inform. Assoc..

[45] Archaya S.D., Elci O.U., Sereika S.M., Styn M.A., Burke L.E. (2011). Using a personal digital assistant for self-monitoring influences diet quality in comparison to a standard paper record among overweight and obese adults. J. Am. Diet Assoc..

[46] Conroy M.B., Yang K., Elci O.U., Gabrielle K.P., Styn M.A., Wang J., Kriska A.M., Sereika S.M., Burke L.E. (2011). Physical activity self-monitoring and weight loss: 6 month results of the SMART trial. Med. Sci. Exerc..

[47] McGough J.M., Faraone S. (2009). Estimating the size of treatment effects. Moving beyond *p* values. Psychiatry.

[48] Michie S., Prestwich A., de Bruin M. (2013). Importance of the nature of the comparison conditions for testing theory-based interventions: Reply. Health Psychol..

[49] Turner-McGrievy G., Kalyanaram S., Campbell K. (2013). Delivering health information via podcast or web: Media effects on psychosocial and physiological respnses. Health Commun..

[50] Webb T.L., Joseph J., Yardley L., Michie S. (2010). Using the internet to promote health behaviour change: A systematic review and meta-analysis of the impact of theoretical basis, use of behaviour change techniques, and mode of delivery on efficacy. J. Med. Internet Res..

[51] (2013). Emedicine. Barriers to Healthy Eating. http://www.emedicinehealth.com/healthy_eating-health/page5_em.htm.

[52] Abraham C., Michie S., Whittington C., McAteer J. (2008). Specifying self-regulation intervention techniques in the context of healthy eating. Int. J. Psychol..

[53] Lyzwinski L.N. (2013). An examination of obesity and eating disorder prevention programmes in schools. Educ. Health.

[54] Dalhgren M., Whitehead M. (2001). developing the policy response to inequities in health. A global perspective. Challenging Inequities in Healthcare: From Ethics to Action.

[55] Adams S. Tax unhealthy foods or else half will be obese by 2030. http://www.telegraph.co.uk/health/8722709/Tax-unhealthy-foods-or-half-will-be-obese-by-2030.html/.

[56] Public Health England (2013). Parks and green spaces. http://www.noo.org.uk/LA/tackling/greenspace.

[57] Marteau T., Olgivie D., Roland M., Suhrcke M., Kelly M.P. (2011). Judging nudging: Can nudging improve population health?. Br. Med. J..

